# The BBSome Controls Energy Homeostasis by Mediating the Transport of the Leptin Receptor to the Plasma Membrane

**DOI:** 10.1371/journal.pgen.1005890

**Published:** 2016-02-29

**Authors:** Deng-Fu Guo, Huxing Cui, Qihong Zhang, Donald A. Morgan, Daniel R. Thedens, Darryl Nishimura, Justin L. Grobe, Val C. Sheffield, Kamal Rahmouni

**Affiliations:** 1 Department of Pharmacology, University of Iowa, Iowa City, Iowa, United States of America; 2 Department of Pediatrics, University of Iowa, Iowa City, Iowa, United States of America; 3 Department of Radiology, University of Iowa, Iowa City, Iowa, United States of America; 4 Fraternal Order of Eagles Diabetes Research Center, University of Iowa, Iowa City, Iowa, United States of America; 5 Department of Internal Medicine, University of Iowa, Iowa City, Iowa, United States of America; The Hospital for Sick Children, CANADA

## Abstract

Bardet-Biedl syndrome (BBS) is a highly pleiotropic autosomal recessive disorder associated with a wide range of phenotypes including obesity. However, the underlying mechanism remains unclear. Here, we show that neuronal BBSome is a critical determinant of energy balance through its role in the regulation of the trafficking of the long signaling form of the leptin receptor (LRb). Targeted disruption of the BBSome by deleting the *Bbs1* gene from the nervous system causes obesity in mice, and this phenotype is reproduced by ablation of the *Bbs1* gene selectively in the LRb-expressing cells, but not from adipocytes. Obesity developed as a consequence of both increased food intake and decreased energy expenditure in mice lacking the *Bbs1* gene in LRb-expressing cells. Strikingly, the well-known role of BBS proteins in the regulation of ciliary formation and function is unlikely to account for the obesogenic effect of BBS1 loss as disruption of the intraflagellar transport (IFT) machinery required for ciliogenesis by deleting the *Ift88* gene in LRb-expressing cells caused a marginal increase in body weight and adiposity. Instead, we demonstrate that silencing BBS proteins, but not IFT88, impair the trafficking of the LRb to the plasma membrane leading to central leptin resistance in a manner independent of obesity. Our data also demonstrate that postnatal deletion of the *Bbs1* gene in the mediobasal hypothalamus can cause obesity in mice, arguing against an early neurodevelopmental origin of obesity in BBS. Our results depict a novel mechanism underlying energy imbalance and obesity in BBS with potential implications in common forms of human obesity.

## Author Summary

The brain plays an important role in maintaining energy homeostasis. The hormone leptin is a critical afferent signal in metabolic homeostasis through its action in the brain. Here we show that neuronal Bardet-Biedl syndrome (BBS) proteins, encoded by genes that cause obesity when mutated, govern energy homeostasis through the control of cell surface expression of the leptin receptor. Selective disruption of BBS proteins causes obesity in mice and impairs the transport of the leptin receptor to the plasma membrane leading to leptin resistance in a manner independent of obesity. These results establish BBS proteins as a fundamental mechanism underlying transport of the leptin receptor and explain why BBS patients develop obesity.

## Introduction

Like many other medical conditions, monogenic obesity syndromes have been invaluable in understanding the biological bases of body weight regulation and dissection of the processes underlying excess fat stores [1,2]. Bardet-Biedl syndrome (BBS) is a highly pleiotropic autosomal recessive disorder in which obesity is a predominant presenting feature [3,4]. Other primary clinical features displayed by BBS patients are retinopathy, polydactyly, learning disabilities and hypogenitalism. BBS is genetically heterogenous with 20 genes (*BBS1-BBS20*) identified to date [5], with multiple protein–protein interactions occurring between the encoded proteins [6]. Indeed, 8 BBS proteins (BBS1, BBS2, BBS4, BBS5, BBS7, BBS8, BBS9 and BBS18 [also known as BBIP10]) form a stable complex, the BBSome, which mediates protein trafficking to the ciliary membrane and perhaps to other membrane compartments [7,8]. BBS3 is a small Ras GTPase and controls BBSome recruitment to the membrane and BBSome ciliary entry [9]. Three other BBS proteins (BBS6, BBS10 and BBS12) form another complex with CCT/TRiC family of group II chaperonins (termed chaperonin-like proteins, CLP) and mediate BBSome assembly [10]. The discovery that various BBS proteins interact to form complexes is consistent with the overlapping phenotypes arising from mutations in different *BBS* genes.

BBS proteins have been known to play a prominent role in the regulation of cilia formation and function by coordinating movement rates of different particles of intraflagellar transport (IFT) machinery which moves cargo proteins within the cilium [11,12]. The importance of BBS proteins for ciliary function is further supported by the fact that *BBS* genes are conserved in ciliated organisms, but not in non-ciliated organisms [13,14]; the action of BBSome as a membrane coat complex that recruits resident proteins to cilia [9]; and the ciliary defects associated with loss of *BBS* genes [15]. Cilia are hair-like cellular projections present in virtually all cell types of the mammalian body [4,16]. These evolutionarily conserved organelles play a fundamental role in the regulation of several biological processes. A wide spectrum of human genetic disorders including BBS, termed ciliopathies, has been attributed to ciliary defects [16]. It should be noted, however, that obesity is not universally associated with ciliopathies [4,17].

BBS mouse models, which present many of the features found in BBS patients including obesity, have been useful in gaining insights into the etiology of this syndrome [15,18–21]. We previously determined that obesity in BBS mutant mice (Bbs1^M390R^, Bbs2^-/-^, Bbs4^-/-^ and Bbs6^-/-^) was associated with hyperphagia and leptin resistance [15,21]. We studied the mechanism of leptin resistance in BBS mutant mice by normalizing their body weight and circulating leptin levels by calorie-restriction [22]. Despite attaining normal serum leptin levels, leptin failed to reduce food intake or activate hypothalamic leptin receptor (LRb) signaling such as Stat3 (signal transducer and activator of transcription-3) [22]. This impairment was accompanied by decreased hypothalamic expression of anorexigenic *proopiomelanocortin (Pomc)* gene without alterations in the expression of orexigenic *agouti protein-related protein (Agrp)* or *neuropeptide y (Npy)* genes [21,22].

Here, we show that disruption of BBS proteins selectively in the nervous system, hypothalamus or LRb-expressing cells, but not in adipocytes, is sufficient to cause obesity. Remarkably, the obesogenic effect of disrupting BBS proteins is unrelated to cilia as interference with ciliogenesis, by disruption of IFT, in LRb cells caused a minimal weight gain. Mechanistically, we demonstrate that silencing the expression of BBS proteins, but not IFT, reduce the surface expression of the LRb leading to leptin resistance independently from obesity.

## Results

### Mice lacking the *Bbs1* gene in the nervous system develop obesity

**Fig 1:**
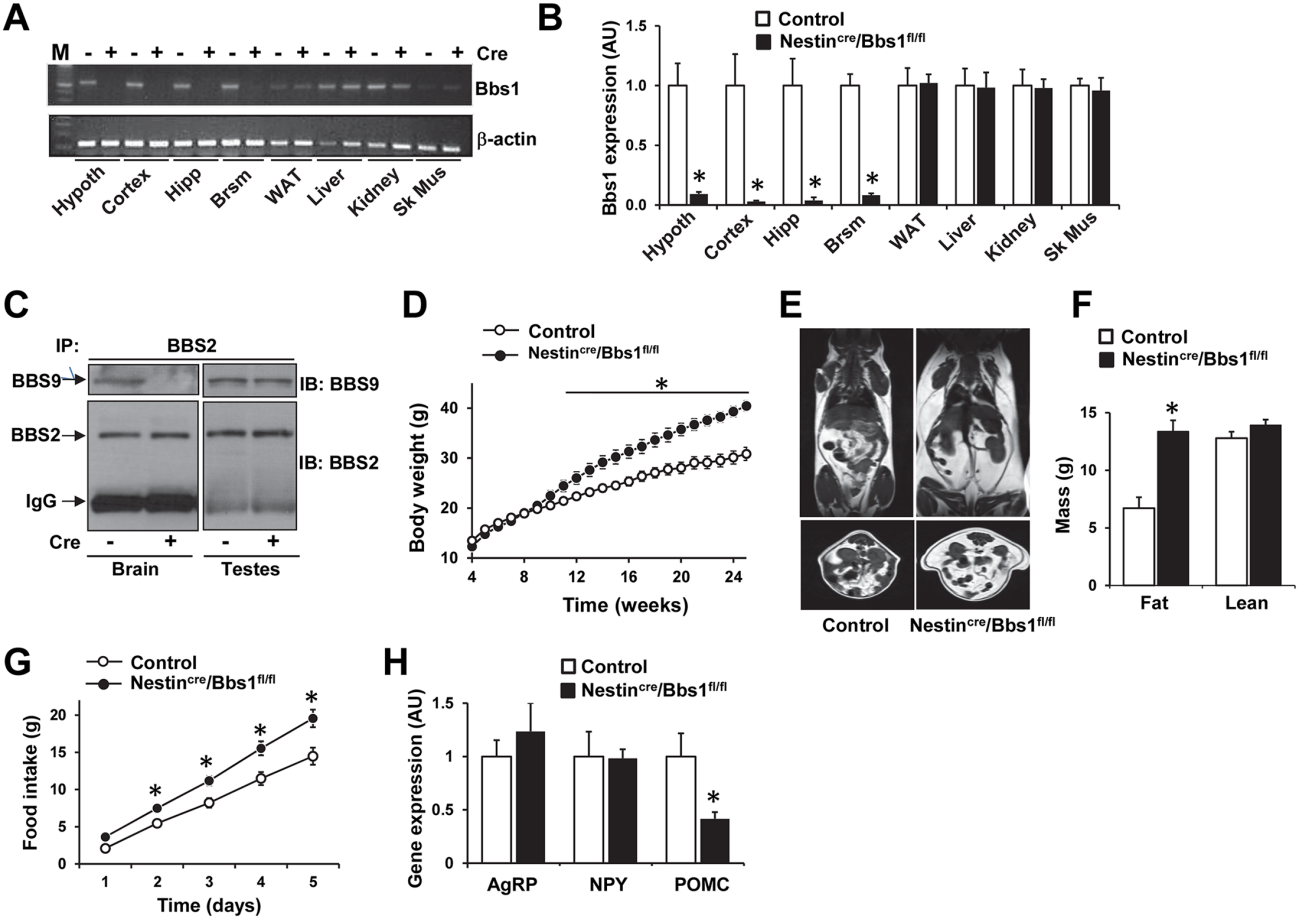
Mice lacking the *Bbs1* gene in the nervous system develop obesity. (A-B) Selectivity of *Bbs1* gene deletion to the nervous system in Nestin^Cre^/Bbs1^fl/fl^ mice as determined by RT-PCR (A) and quantitative RT-PCR (B, n = 1 male and 2 females in group). *Bbs1* expression was absent in the brain (hypothalamus [Hypoth], cortex, hippocampus [Hippo] and brainstem [Brsm]) of Nestin^Cre^/Bbs1^fl/fl^ mice (Cre+) relative to controls (Cre-). However, *Bbs1* expression was not altered in white adipose tissue (WAT), liver, kidney and skeletal muscle (Sk Mus). (C) Analysis of the BBSome complex in the brain and testes of Nestin^cre^/Bbs1^fl/fl^ mice. Inability of BBS2 to pull down BBS9 in the brain, but not in the testes, of Nestin^Cre^/Bbs1^fl/fl^ mice (Cre+) relative to controls (Cre-). (D) Weekly body weights of Nestin^cre^/Bbs1^fl/fl^ and control mice (n = 12 males and 12 females for controls; 6 males and 6 females for Nestin^cre^/Bbs1^fl/fl^ mice). (E-F) Comparison of body composition between Nestin^cre^/Bbs1^fl/fl^ and control mice. Shown are coronal (top) and axial abdominal (bottom) representative MRI sections (E) and total fat mass and lean mass (F) of 25 weeks old control and Nestin^cre^/Bbs1^fl/fl^ mice (n = 6 males and 6 females in each group). (G) Cumulative food intake of Nestin^cre^/Bbs1^fl/fl^ and littermate controls (n = 6 males and 6 females for controls, and 6 males and 8 females for Nestin^cre^/Bbs1^fl/fl^ mice). (H) Hypothalamic mRNA levels of AgRP, NPY and POMC in Nestin^cre^/Bbs1^fl/fl^ and control mice (n = 3 males and 3 females in each group). Data are means ± SEM, *P< 0.05 vs control group.

To gain insight into the role of BBS proteins in the nervous system in the regulation of energy homeostasis we used a conditional knockout mouse model where exon 3 of the *Bbs1* gene is floxed [23]. Breeding Bbs1^fl/fl^ and Nestin^Cre^ mice created mice deficient in *Bbs1* gene only in the nervous system as indicated by the loss of *Bbs1* gene expression in the brain, but not in peripheral tissues (Fig 1A and 1B). Moreover, using co-immunoprecipitation assays we found that Nestin^Cre^/Bbs1^fl/fl^ mice have disrupted BBSome in the nervous system as indicated by the inability of BBS2 to pull down BBS9 in the brain whereas in testes the interaction between BBS2 and BBS9 was not altered (Fig 1C) indicating that in Nestin^Cre^/Bbs1^fl/fl^ mice disruption of the BBSome was specific to the nervous system.

From the time of weaning (4 weeks) up to 8 weeks of age, Nestin^Cre^/Bbs1^fl/fl^ mice weighed the same as the littermate controls. However, by about 9 weeks of age, Nestin^Cre^/Bbs1^fl/fl^ mice began gaining significantly more weight than the littermate controls (Fig 1D). Notably, the obesity phenotype was more pronounced in the females relative to males (S1A and S1B Fig). At 25 weeks of age, the female Nestin^Cre^/Bbs1^fl/fl^ mice weighed about 53% more than the controls while the male Nestin^Cre^/Bbs1^fl/fl^ mice weighed about 18% more than their littermate controls.

The increased body weight in Nestin^Cre^/Bbs1^fl/fl^ mice was associated with substantial increase in fat mass as measured by MRI in 12- and 25-week old mice (Fig 1E and 1F). This was further confirmed by weighing individual fat pads at sacrifice (S1C Fig). However, there was no difference in lean mass as determined by MRI (Fig 1F). Consistent with the sex difference in body weight, the increased weight of fat pads in Nestin^Cre^/Bbs1^fl/fl^ mice was more pronounced in females relative to males (S1D and S1E Fig). These findings are consistent with what is observed in the mice bearing global deletion of *BBS* genes.

Feeding studies indicated that Nestin^Cre^/Bbs1^fl/fl^ mice are hyperphagic. Measurement of food intake during 5 days showed that obese Nestin^Cre^/Bbs1^fl/fl^ mice eat about 35% more than the controls (Fig 1G). We also examined the gene expression profile of the key hypothalamic orexigenic and anorexigenic neuropeptides involved in the regulation of energy homeostasis. We found that mRNA levels of AgRP and NPY were normal, whereas the mRNA level of POMC was significantly reduced in Nestin^Cre^/Bbs1^fl/fl^ mice (Fig 1H). Together, these findings demonstrate the importance of *Bbs1* gene in the nervous system for energy homeostasis and the obesity phenotype of BBS.

Next, we tested whether the obesity phenotype can be recapitulated by deleting the *Bbs1* gene in tissues other than the nervous system. In cultured adipocytes, inactivation of *BBS* genes accelerated cell division, caused aberrant differentiation and promoted adipogenesis and fat accumulation [24,25] pointing to a possible contribution of adipose tissue to BBS-associated obesity. Thus, we crossed the Bbs1^fl/fl^ mice with Adiponectin (Adip)^Cre^ mice in which Cre expression is restricted to adipocytes [26]. However, there was no difference in body weight, fat pad weights or adipocytes size between Adip^Cre^/Bbs1^fl/fl^ mice and littermate controls (S2A–S2D Fig). These findings indicate that the changes that occur in adipocytes lacking *BBS* genes *in vitro* may not translate *in vivo*. Alternatively, it is possible that early loss of the *Bbs1* gene in adipocytes may have caused compensatory adaptations that protect the mice from metabolic alterations and obesity. This is unlikely given that lifelong *Bbs1* gene deficiency in the nervous system caused obesity.

### Deleting the *Bbs1* gene in the LRb-expressing cells causes obesity

**Fig 2:**
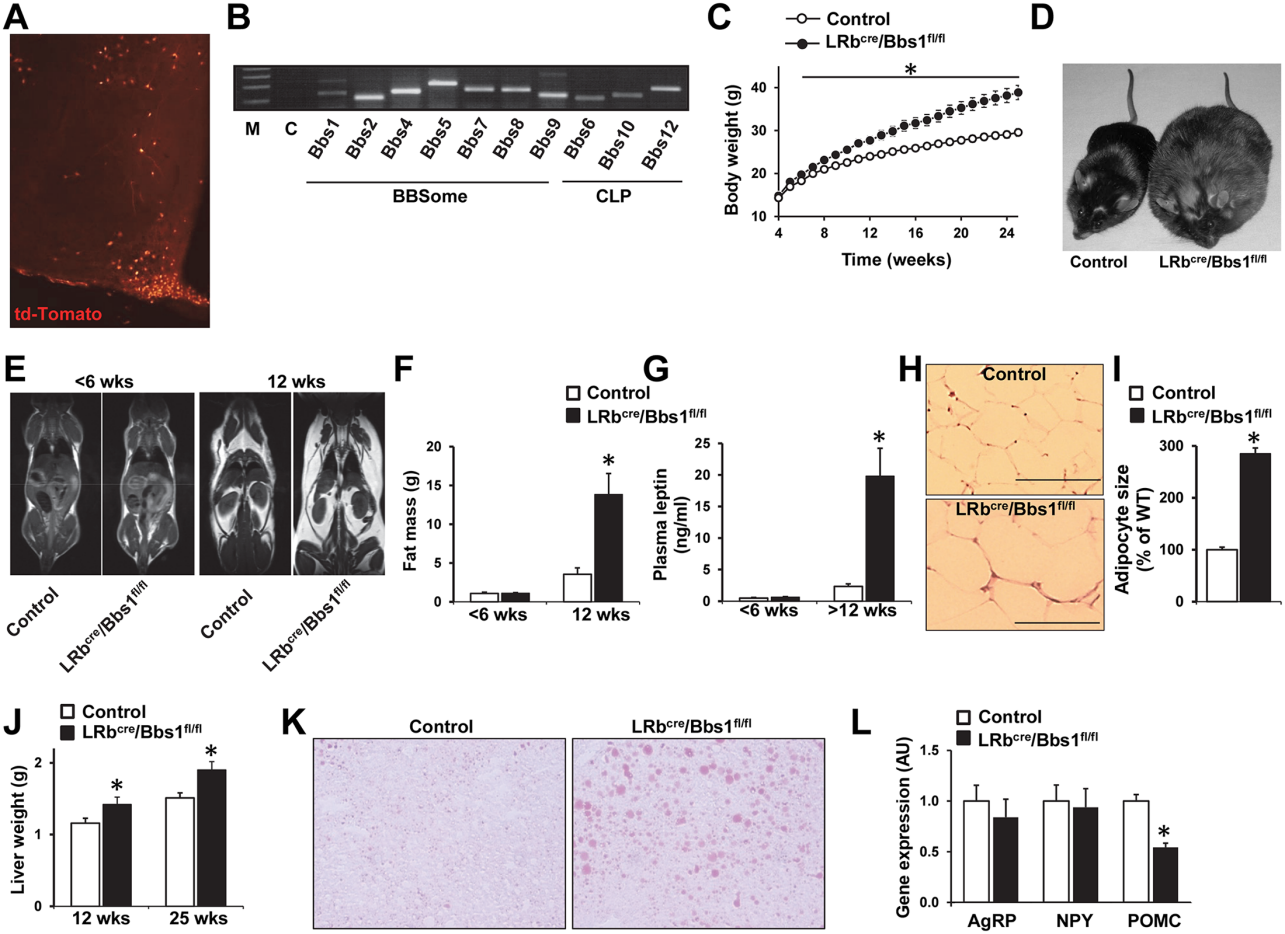
Relevance of the *Bbs1* gene in LRb-containing cells for body weight regulation. (A) Visualization of Cre-mediated recombination in the hypothalamus using the td-Tomato reporter mice crossed with LRb^cre^ mice. (B) Presence of the genes encoding the BBSome and CLP proteins in the FACS-sorted LRb labeled cells as determined by RT-PCR. (C) Weekly body weights of LRb^cre^/Bbs1^fl/fl^ mice and littermate controls (n = 13 males and 14 females for controls, and 11 males and 11 females for LRb^cre^/Bbs1^fl/fl^ mice). (D) A photograph showing morbid obesity in a 9 month old LRb^cre^/Bbs1^fl/fl^ mouse relative its littermate control. (E) Representative MRI images of young (<6 weeks of age) and older (12 weeks of age) LRb^cre^/Bbs1^fl/fl^ mice and their littermate controls. (F) Total fat mass of young (<6 weeks of age) and old (12 weeks of age) LRb^cre^/Bbs1^fl/fl^ mice and their littermate controls (n = 3 males and 6 females in each group). (G) Plasma leptin levels of of young (<6 weeks of age) and older (>12 weeks of age) LRb^cre^/Bbs1^fl/fl^ mice and their littermate controls (n = 6 males and 6–16 females for control, and 2–7 males and 7 females for LRb^cre^/Bbs1^fl/fl^ mice). (H—I) Representative HE staining (H) and quantification of the mean adipocyte size (I) of peri-gonadal fat pad of 12 weeks old LRb^cre^/Bbs1^fl/fl^ mice and controls (n = 3 males and 3 females in each group). (J) Liver weights of 12 and 25 weeks old LRb^cre^/Bbs1^fl/fl^ mice and controls (n = 3 males and 3 females in each group). (K) Representative Oil-Red-O liver staining of 25 weeks old LRb^cre^/Bbs1^fl/fl^ and control mice. (L) mRNA levels of AgRP, NPY and POMC in the hypothalamus of LRb^cre^/Bbs1^fl/fl^ and control mice (n = 3 males and 3 females in each group). Data are means ± SEM, *P< 0.05 vs. control group.

The influential role of leptin in the regulation of energy homeostasis [27] led us to ask whether deletion of the *Bbs1* gene specifically in the LRb-expressing cells will recapitulate the obesity phenotype of BBS. For this, we used LRb^Cre^ mice which provide specificity of Cre expression to LRb expressing cells (see Fig 2A and Refs [28,29]). First, we assessed whether LRb cells express *Bbs* genes. We used Fluorescence Activated Cell Sorting (FACS) procedure [30,31] to isolate LRb-positive cells (S3 Fig). We found that FACS-purified LRb containing cells (td-Tomato^+^) of the hypothalamus express all the *Bbs* genes tested including those encoding proteins of the BBSome (*Bbs1*, *2*, *4*, *5*, *7*, *8* and *9*) and the CLP complex (*Bbs6*, *10* and *12*) (Fig 2B).

At 4 and 5 weeks of age, there was no difference in body weight between LRb^Cre^/Bbs1^fl/fl^ mice and littermate controls (Fig 2C). At around 6 weeks of age, LRb^Cre^/Bbs1^fl/fl^ mice start gaining more weight than the controls. The divergence in body weight continues as the mice grow (Fig 2C). At 25 weeks of age, the LRb^Cre^/Bbs1^fl/fl^ mice weighed about 31% more than the controls (Fig 2C) and become morbidly obese as they age (Fig 2D). In contrast to the Nestin^Cre^/Bbs1^fl/fl^ mice, the weight gain trend was similar in male and female LRb^Cre^/Bbs1^fl/fl^ mice relative to their littermate controls (S4A and S4B Fig).

Fat mass and plasma leptin levels were not different in young (<6-week old) LRb^Cre^/Bbs1^fl/fl^ mice relative to controls (Fig 2E–2G), but older LRb^Cre^/Bbs1^fl/fl^ mice develop adiposity as indicated by the increased fat mass (Figs 2E, 2F and S4C and S4D), plasma leptin (Fig 2G) and size of adipocytes (Fig 2H and 2I). Liver mass was also increased in LRb^Cre^/Bbs1^fl/fl^ mice (Fig 2J) which was associated with accumulation of fat as revealed by Oil-Red-O staining (Fig 2K). Similar to the Nestin^Cre^/Bbs1^fl/fl^ mice, the LRb^Cre^/Bbs1^fl/fl^ mice exhibited normal expression of hypothalamic orexigenic *Agrp* and *Npy* genes while the expression of anorexigenic *Pomc* gene was significantly reduced (Fig 2L). Interestingly, hypothalamic *Pomc* gene expression was also decreased in young LRb^Cre^/Bbs1^fl/fl^ mice (0.56±0.07 AU vs 1.00±0.14 AU in controls, P = 0.007). Of note, *Bbs1* gene deletion did not appear to affect cilia in LRb expressing neurons (S4E Fig). Collectively, these findings highlight the importance of *BBS* genes in LRb-containing cells for body weight regulation in a manner independent of cilia.

### Energy imbalance in mice lacking the *Bbs1* in LRb-expressing cells

**Fig 3:**
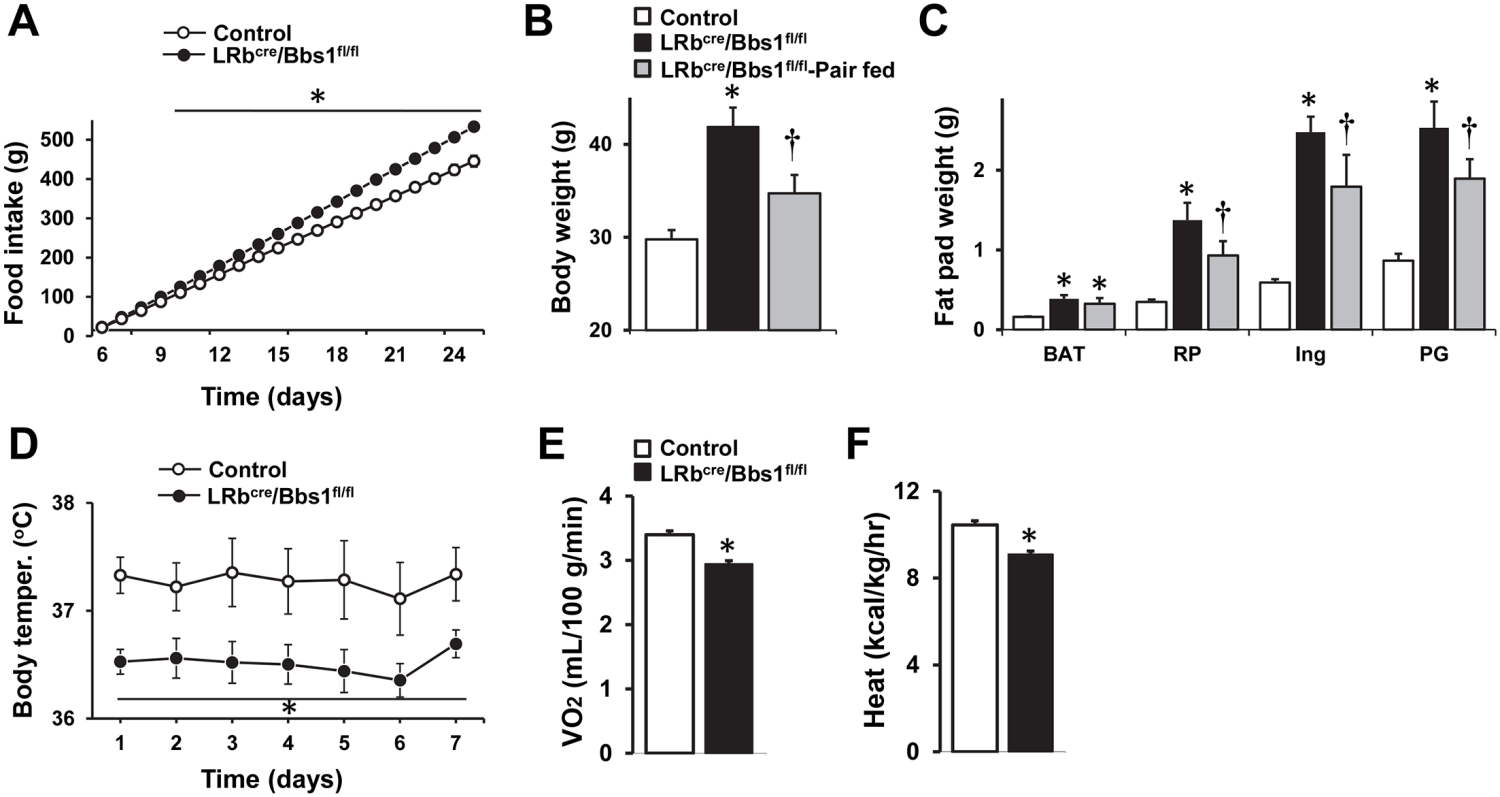
Energy imbalance leading to obesity in LRb^cre^/Bbs1^fl/fl^ mice. (A) Cumulative weekly food intake of LRb^cre^/Bbs1^fl/fl^ mice compared with littermate controls (n = 12 males and 10 females for controls and 7 males and 4 females for LRb^cre^/Bbs1^fl/fl^ mice). (B-C) Average body weights and fat pad weights (brown adipose tissue [BAT], retro-peritoneal [RP], Inguinal [Ing)] and peri-gonadal [PG]) of 25 weeks old pair-fed LRb^cre^/Bbs1^fl/fl^ mice relative to controls and LRb^cre^/Bbs1^fl/fl^ mice fed *ad libitum* (n = 3 males and 3 females in each group). (D) Average 24-hour core body temperatures of LRb^cre^/Bbs1^fl/fl^ and control mice. (n = 2 males and 3 females in each group). (E–F) Energy expenditure as determined by VO_2_ (E) and heat generation (F) in weight-matched 8–10 weeks old LRb^cre^/Bbs1^fl/fl^ mice and littermate controls (n = 3 males and 3 females in each group). Data are means ± SEM, *P< 0.05 vs. control group, †P<0.05 vs. controls and LRb^cre^/Bbs1^fl/fl^ mice fed *ad libitum*.

Food intake was no different between LRb^Cre^/Bbs1^fl/fl^ and control mice at 4 (P = 0.4), 5 (P = 0.35) and 6 weeks (P = 0.22) of age. However, after 6 weeks of age, the LRb^Cre^/Bbs1^fl/fl^ mice eat consistently more than the controls (Fig 3A). Cumulatively, the LRb^Cre^/Bbs1^fl/fl^ mice eat about 20% more than the controls. Bomb calorimetric analyses of fecal caloric content showed that LRb^Cre^/Bbs1^fl/fl^ mice have normal digestive efficiency (absorbing 79±1% vs. 78±1% in control mice, P = 0.20), indicating that the elevated food intake of the LRb^Cre^/Bbs1^fl/fl^ mice translate into an increase in total caloric absorption and availability.

To examine whether hyperphagia can explain the increased adiposity in LRb^Cre^/Bbs1^fl/fl^ mice, we performed a pair-feeding study by providing each LRb^Cre^/Bbs1^fl/fl^ mouse the same amount of food eaten daily by the littermate control beginning at 5 weeks of age. At 25 weeks, body weight and fat pad masses of pair-fed LRb^Cre^/Bbs1^fl/fl^ mice were substantially lower relative to the LRb^Cre^/Bbs1^fl/fl^ mice fed *ad libitum* (Fig 3B and 3C) indicating that hyperphagia contribute to the obesity phenotype of LRb^Cre^/Bbs1^fl/fl^ mice. However, body weight and fat pad masses of the 25 weeks old pair-fed LRb^Cre^/Bbs1^fl/fl^ mice remained significantly elevated compared to age-matched littermate controls (Fig 3B and 3C). Similar trend was observed in a subset of LRb^Cre^/Bbs1^fl/fl^ mice that were pair-fed until 15 weeks of age (S5A and S5B Fig). These findings suggest that LRb^Cre^/Bbs1^fl/fl^ mice have reduced energy expenditure. In favor of such possibility, 24-h body temperature, measured by radiotelemetry during 7 days, was consistently lower in the LRb^Cre^/Bbs1^fl/fl^ mice as compared to the controls (Fig 3D).

Next, we used indirect calorimetry to assess energy expenditure in young weight-matched LRb^Cre^/Bbs1^fl/fl^ mice (23.7±1.2 g) and littermate controls (23.2±0.3 g). Both O_2_ consumption (Fig 3E) and heat production (Fig 3F) were significantly lower in the obese LRb^Cre^/Bbs1^fl/fl^ mice relative to controls. Young LRb^Cre^/Bbs1^fl/fl^ mice tend to have lower O_2_ consumption (2.68±0.17 ml/100g/min) and heat production (8.04±0.47 kcal/kg/h) relative to age-matched controls (2.92±0.07 ml/100g/min and 8.53±0.20 kcal/kg/h, respectively), but this was not statistically different (P = 0.08 and 0.16, respectively). Together, these findings demonstrate that both hyperphagia and decreased energy expenditure contribute to the obesity phenotype of the LRb^Cre^/Bbs1^fl/fl^ mice. Of note, Nestin^Cre^/Bbs1^fl/fl^ mice (weight-matched to littermate controls) also exhibit a significantly reduced O_2_ consumption and heat production (S5C and S5D Fig).

### Leptin resistance in LRb^Cre^/Bbs1^fl/fl^ mice is independent of obesity

The presence of obesity and hyperleptinemia in the LRb^Cre^/Bbs1^fl/fl^ mice prompted us to test whether these mice are leptin resistant. We found that intraperitoneal (i.p.) administration of leptin (1 μg/g body weight, twice daily) was less effective in reducing food intake and body weight in obese LRb^Cre^/Bbs1^fl/fl^ mice relative to controls (S6A and S6B Fig).

**Fig 4:**
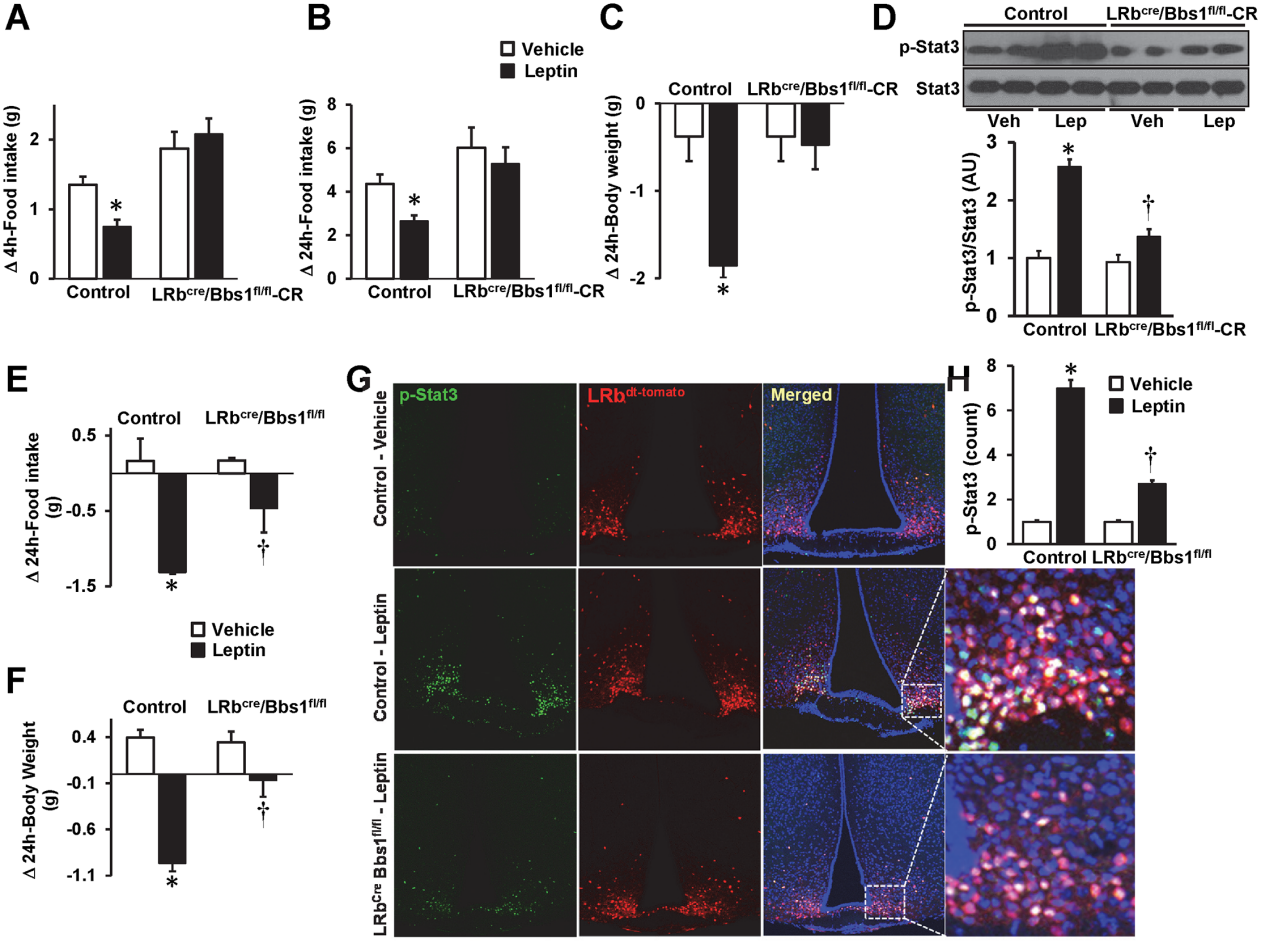
Leptin resistance in LRb^cre^/Bbs1^fl/fl^ mice is not related to obesity. (A-C) Effect of i.c.v. administration of leptin (2 μg) relative to vehicle on 4- (A) and 24-hour (B) food intake and 24-hour body weight (C) in 14–16 weeks old calorie restricted (CR) LRb^cre^/Bbs1^fl/fl^ mice compared to controls (n = 2 males and 3 females in each group). (D) Western blot analysis of i.c.v. leptin (2 μg)-induced phospho-Stat3 increase in the mediobasal hypothalamus of 14–16 weeks old CR LRb^cre^/Bbs1^fl/fl^ mice and controls (n = 2 males and 2 females in each group). (E–F) Effect of i.p. administration of leptin (1 μg/g bw, twice a day) on 24-hour food intake (E) and body weight (F) in young (<6 weeks old) LRb^cre^/Bbs1^fl/fl^ mice and controls (n = 4 males and 4 females for controls and 5 males and 5 females for LRb^cre^/Bbs1^fl/fl^ mice). (G-H) Confocal images (G) and quantification data (H) depicting the effect of i.p. leptin (2 μg/g bw) on phospho-Stat3 in the hypothalamic arcuate nucleus of young (<6 weeks old) LRb^cre^/Bbs1^fl/fl^ mice and controls (n = 2 males and 3 females in each group). Data are means ± SEM, *P< 0.05 vs. control-vehicle and †P<0.05 vs. control-leptin group.

To test the contribution of obesity to leptin resistance in LRb^Cre^/Bbs1^fl/fl^ mice, we performed several experiments. First, we tested leptin sensitivity in LRb^Cre^/Bbs1^fl/fl^ mice that remained lean throughout their lifespan. For this, beginning at weaning we restricted the amount of food LRb^Cre^/Bbs1^fl/fl^ mice could eat (by feeding them 70–80% of daily amount of food eaten by the littermate controls) so that they could not become obese. This strategy prevented the development of obesity in the LRb^Cre^/Bbs1^fl/fl^ mice as indicated by the normalization of plasma leptin levels (3.0±0.3 ng/mL in calorie restricted LRb^Cre^/Bbs1^fl/fl^ mice vs. 3.1±0.5 ng/mL in control mice). Calorie-restricted LRb^Cre^/Bbs1^fl/fl^ mice were resistant to the anorectic and weight-reducing effects induced by intracerebroventricular (i.c.v.) injection of leptin (Fig 4A–4C). Moreover, leptin-evoked activation of hypothalamic Stat3 (p-Stat3) was significantly reduced in the calorie-restricted LRb^Cre^/Bbs1^fl/fl^ mice as assessed by Western blotting (Fig 4D).

We also tested leptin sensitivity in young LRb^Cre^/Bbs1^fl/fl^ mice before the development of obesity and hyperleptinemia (Fig 2E–2G). Treatment with i.p. leptin decreased food intake and body weight in the young LRb^Cre^/Bbs1^fl/fl^ mice, but these responses were substantially attenuated compared to the control mice (Fig 4E and 4F). We also used immunohistochemistry to assess Stat3 activation in the arcuate nucleus of young LRb^Cre^/Bbs1^fl/fl^ mice which were bred with the td-Tomato reporter mice to fluorescently label the LRb-expressing cells. Relative to controls, young LRb^Cre^/Bbs1^fl/fl^ mice displayed a substantially reduced leptin-induced hypothalamic Stat3 activation by immunohistochemistry (Fig 4G and 4H). Collectively, these data demonstrate that leptin resistance in mice lacking the *Bbs1* gene in the LRb-expressing cells is not secondary to obesity.

We considered the possibility that inactivation of the *Bbs1* gene may alter the expression of the LRb or number of LRb-expressing cells leading to leptin resistance. However, the mRNA level of the LRb in the hypothalamic explants was not significantly different in the LRb^Cre^/Bbs1^fl/fl^ mice (1.38±0.7 AU) relative to the controls (1.00±0.2 AU, p = 0.3) indicating that leptin resistance in the LRb^Cre^/Bbs1^fl/fl^ mice is not due to decreased *LRb* gene expression. The number of LRb-expressing cells in the hypothalamic arcuate nucleus was also comparable between the LRb^Cre^/Bbs1^fl/fl^ mice and controls (S6C Fig). Alternatively, leptin resistance can be caused by overabundance of the negative feedback inhibitors of the LRb signaling such as the suppressor of cytokine signaling 3 (SOCS3) [32] or the protein-tyrosine phosphatase 1B (PTP1B) [33]. We found that levels of hypothalamic SOCS3 mRNA were not altered while the mRNA level of PTP1B was reduced in the LRb^Cre^/Bbs1^fl/fl^ animals (S6D Fig) excluding these negative inhibitors of LRb signaling as a mechanism of leptin resistance in the LRb^Cre^/Bbs1^fl/fl^ mice. We also investigated the potential role of the LR gene-related protein (LRGRP) a negative regulator of LRb transport to the cell surface and previously implicated in the development of leptin resistance in diet-induced obesity [34]. However, the mRNA levels of LRGRP tended to be lower in the hypothalamic explants of the LRb^Cre^/Bbs1^fl/fl^ mice relative to littermate controls (S6D Fig).

### Postnatal hypothalamic disruption of the *Bbs1* gene promotes adiposity

**Fig 5:**
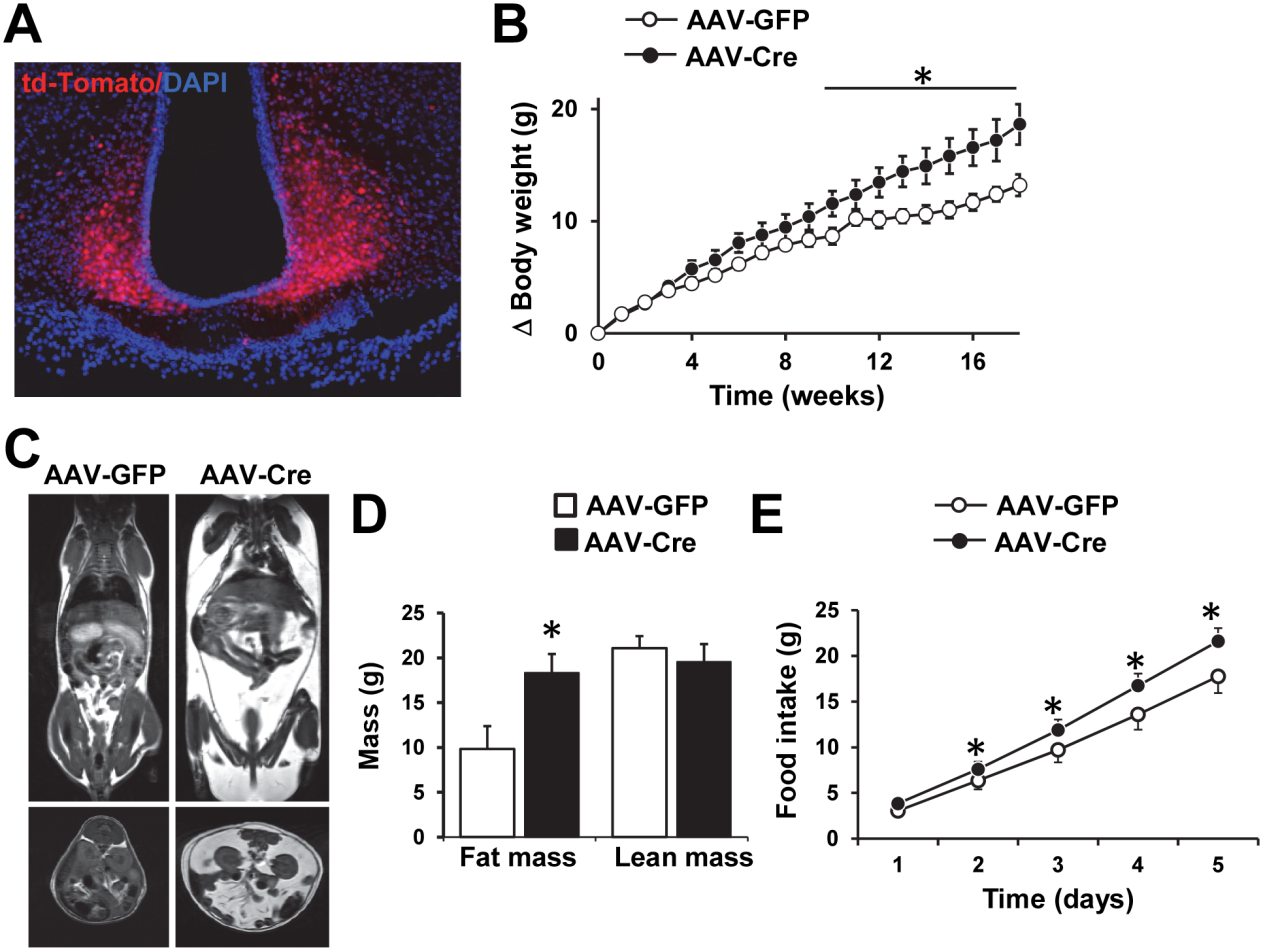
Postnatal deletion of the *Bbs1* gene in the mediobasal hypothalamus increases weight gain and adiposity. (A) Cre-mediated recombination in the mediobasal hypothalamus after AAV-Cre microinjection was visualized by td-Tomato expression in Bbs1^fl/fl^/td-Tomato mice. (B) Average weekly body weight changes after bilateral microinjection of AAV-GFP or AAV-Cre into the mediobasal hypothalamus of Bbs1^fl/fl^/td-Tomato mice (n = 8 females per group). (C-E) Representative MRI images (C), body composition (D) and cumulative 5 days food intake (E) of Bbs1^fl/fl^/td-Tomato mice received bilateral injection of AAV-GFP or AAV-Cre (n = 7 females per group). Data are means ± SEM, *P< 0.05 vs. AAV-GFP group.

We asked whether postnatal deletion of the *Bbs1* gene alters body weight and adiposity. The evidences including the decreased *Pomc* gene expression points to the mediobasal hypothalamus as a potential site underlying energy imbalance in BBS. Thus, we tested the effect on body weight of deleting the *Bbs1* gene in the mediobasal hypothalamus which includes the arcuate and ventromedial nuclei. We stereotaxically targeted the mediobasal hypothalamus bilaterally with an adeno-associated virus expressing Cre (AAV-Cre) using a previously validated strategy [35]. Because td-Tomato/Bbs1^fl/fl^ mice were used in this study, induction of td-Tomato (assessed at the conclusion of the experiments in every animal) was used as an indication of Cre expression (Fig 5A).

Bbs1^fl/fl^ mice (7–10 weeks old) received bilateral microinjections into the mediobasal hypothalamus of either AAV-Cre or an AAV expressing enhanced green fluorescence protein (AAV-GFP), used as controls. Body weight was not different between the 2 groups at baseline or during the first 4–5 weeks after stereotaxic injections (Fig 5B). However, after 5 weeks, Bbs1^fl/fl^ mice treated with AAV-Cre began to gain more weight than the AAV-GFP infused group. After 9 weeks, body weight was consistently elevated in the AAV-Cre injected mice (Fig 5B). Fat mass measured at 18 weeks after stereotaxic injections was significantly elevated in AAV-Cre treated mice relative to AAV-GFP controls (Figs 5C, 5D and S7) whereas lean mass was comparable between the 2 groups (Fig 5D). Measurement of food intake showed that AAV-Cre treated mice eat significantly more than the controls (Fig 5E). Thus, postnatal ablation of the *Bbs1* gene specifically in the mediobasal hypothalamus is sufficient to cause hyperphagia and obesity.

### Ablation of the *Ift88* gene in LRb neurons induces minimal leptin resistance

**Fig 6:**
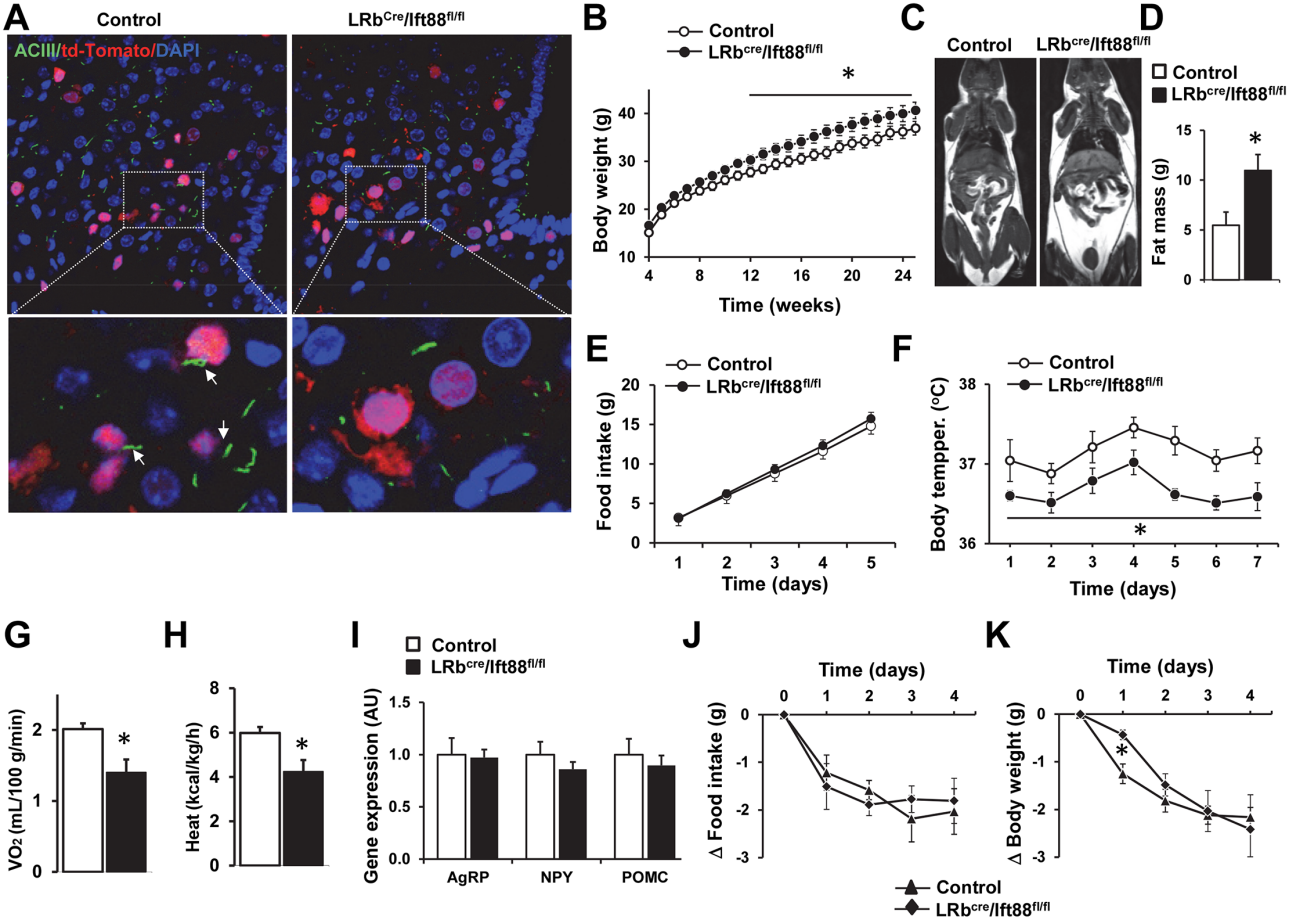
Characterization of mice lacking the *Ift88* gene in LRb cells. (A) Representative confocal images (low and high magnifications) of the hypothalamic arcuate nucleus showing loss of cilia (ACIII immunostaining) in the LRb-positive cells, but not in LRb-negative cells. The arrows point to cilia of LRb-positive cells in the control mouse. (B) Body weights of LRb^cre^/Ift88^fl/fl^ mice and littermate controls (n = 12 males and 15 females for control group and 11 males and 13 females for LRb^cre^/Ift88^fl/fl^ mice). (C-D) Representative MRI images (C) and total fat mass (D) of 25 weeks old LRb^cre^/Ift88^fl/fl^ mice and controls (n = 5 females per group). (E) Cumulative 5 days food intake of 25 weeks LRb^cre^/Ift88^fl/fl^ mice and control littermates (n = 7–8 males and 10 females in each group). (F) Average 24-hour telemetric core body temperatures of LRb^cre^/Ift88^fl/fl^ mice and controls (n = 3 males and 4 females in each group). (G–H) Oxygen consumption (G, VO_2_) and heat generation (H) in LRb^cre^/Ift88^fl/fl^ and control mice (n = 4 males and 4 females in each group). (I) mRNA levels of AgRP, NPY and POMC in the hypothalamus of LRb^cre^/Ift88^fl/fl^ mice and littermate controls (n = 3 males and 3 females in each group). (J–K) Effect of i.p. administration of leptin (1 μg/g bw, twice daily) on food intake (J) and body weight (K) in LRb^cre^/Ift88^fl/fl^ and control mice (n = 3 males and 4–5 females in each group). Data are means ± SEM, *P< 0.05 vs. control group.

To test whether loss of other cilia-related genes in LRb-containing neurons recapitulate the obesity phenotype induced by ablation of the *Bbs1* gene, we crossed the mice bearing the conditional allele of the *Ift88* gene [36] with the LRb^Cre^ mice. As expected, LRb^Cre^/Ift88^fl/fl^ mice displayed loss of cilia (ACIII staining) specifically in the LRb expressing neurons. Indeed, in the LRb^Cre^/Ift88^fl/fl^ mice cilia were unaffected in non-LRb-containing cells whereas virtually no cilia or severely truncated cilia were detected in the LRb expressing cells (Fig 6A), which is consistent with the requirement of IFT for ciliogenesis [11,12].

The LRb^Cre^/Ift88^fl/fl^ mice exhibited a slight, but significantly elevated body weight (Fig 6B) and fat mass (Fig 6C and 6D) than their littermate controls. Of note, the increase in body weight and adiposity was more noticeable in the males than the females LRb^Cre^/Ift88^fl/fl^ mice (S8A–S8D Fig). Food intake was not different between LRb^Cre^/Ift88^fl/fl^ mice and littermate controls (Fig 6E) suggesting that the increased adiposity in LRb^Cre^/Ift88^fl/fl^ mice is not caused by hyperphagia. Rather, a decrease in energy expenditure seems to account for the increased adiposity in LRb^Cre^/Ift88^fl/fl^ mice as indicated by the slight, but significantly lower body temperature (Fig 6F), O_2_ consumption (Fig 6G) and heat production (Fig 6H) in these mice. Hypothalamic expression levels of *Agrp*, *Npy* and *Pomc* genes were not altered in LRb^Cre^/Ift88^fl/fl^ mice (Fig 6I).

Leptin-induced decrease in food intake was indistinguishable between LRb^Cre^/Ift88^fl/fl^ mice and littermate controls during the 4-day treatment (Fig 6J). The decrease in body weight evoked by leptin was significantly attenuated in the LRb^Cre^/Ift88^fl/fl^ mice during the first day, but not during the remaining 3 days of treatment (Fig 6K). These data demonstrate minimal leptin resistance in adult LRb^Cre^/Ift88^fl/fl^ mice which is likely due to the increased adiposity as leptin sensitivity was not altered in lean young LRb^Cre^/Ift88^fl/fl^ mice as indicated by the comparable decrease in food intake and body weight in young (6–8 weeks old) LRb^Cre^/Ift88^fl/fl^ mice relative to controls (S8E and S8F Fig). These findings indicate that leptin resistance does not account for the increased adiposity associated with the disruption of the *Ift88* gene. This implies that LRb-independent mechanisms contribute to the obesity associated with ciliopathies other than BBS.

### BBS1, but not IFT88, is required for the surface expression of the LRb

**Fig 7:**
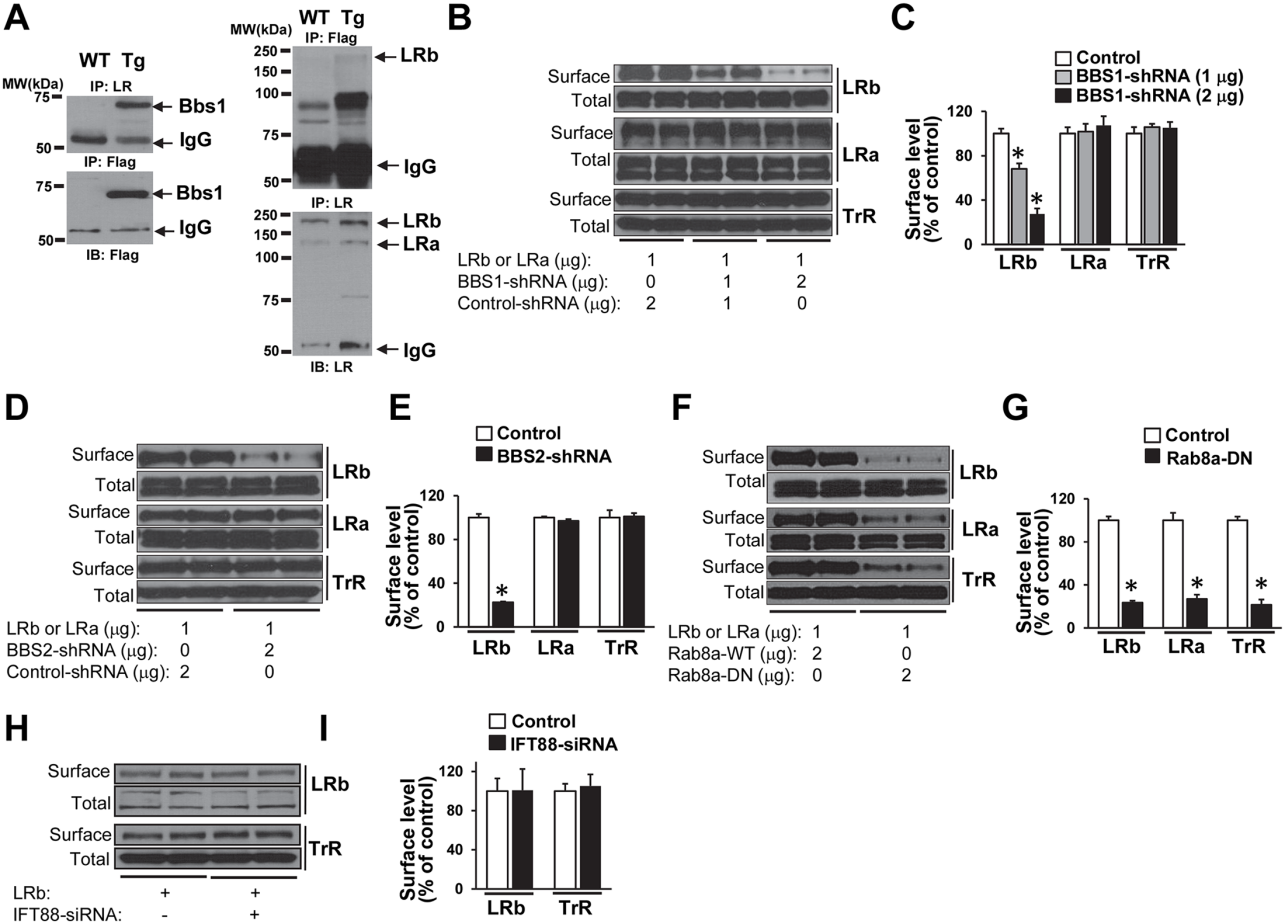
BBS1 protein interacts with the LRb and regulates its trafficking to the cells surface. (A) Interaction between the BBS1 protein and the endogenous LRb in brain lysate. Ability of Flag-tagged BBS1 and LRb to pull down each other by co-immunoprecipitation assays using brain lysates of transgenic (Tg) mice expressing a Flag-BBS1 protein. IP: immunoprecipitation, IB: immunoblot (representative of n = 3, 1 male and 2 females). (B–C) shRNA-mediated silencing of BBS1 reduces the surface levels of Flag-tagged LRb in a dose-dependent manner in HEK 293 cells transiently co-transfected with Flag-LRb and Bbs1-shRNA plasmids. This effect is specific as the surface expression of HA-tagged LRa (in separate experiments) and endogenous transferrin receptor (TrR, in same experiments as the Flag-LRb) were not altered (n = 6 per group). (D-E) Silencing of BBS2, another BBSome protein, lowers the surface levels of Flag-LRb, but not HA-LRa or endogenous TrR in HEK 293 cells (n = 6 per group). (F–G) Blockade of Rab8a using a dominant negative (DN) form decreases the surface expression of Flag-LRb, HA-LRa and endogenous TrR in HEK 293 cells (n = 6 per group). (H–I) siRNA-mediated silencing of Ift88 had no effect on the surface expression of Flag-LRb or endogenous TrR in HEK 293 cells (n = 8 per group). Data are means ± SEM, *P< 0.05 vs. control group.

We considered the possibility that BBS proteins may be involved in transporting the LRb to the cell membrane and that loss of the *Bbs1* gene may cause abnormal trafficking and localization of the LRb. We previously demonstrated that BBS1 protein specifically interacts with the LRb, but not with LRa, in transiently transfected cells [22]. To substantiate this biochemical interaction further *in vivo*, we generated a new transgenic mouse model that expresses a Flag-tagged BBS1 protein. Co-immunoprecipitation assays using an antibody recognizing both the LRb and LRa (S9A Fig) showed that Flag-BBS1 interacts with the endogenous LRb, but not LRa, in protein lysates prepared from transgenic mouse brains (Fig 7A). Of note, Flag-BBS1 and LRb can also be reciprocally immunoprecipitated using hypothalamic protein lysates, but not in protein lysates from the cortex (S9B Fig).

Using HEK-293 cells, we examined whether knockdown of BBS1 expression impacts LRb cell surface expression. Cells were transiently co-transfected with Flag-tagged LRb together with increased dose of BBS1 shRNA (S10A Fig). Flag-LRb cell surface expression was quantified using a surface protein biotinylation assay. We observed a dose-dependent decrease in Flag-LRb cell surface levels in response to BBS1 knockdown (Fig 7B and 7C). In contrast, the total amount of Flag-LRb was not affected. Consistent with the reduced Flag-LRb in the plasma membrane, leptin-activation of Stat3 and S6K was attenuated in the BBS1 knockdown cells compared to control cells (S10B and S10C Fig). These results demonstrate that BBS1 knockdown alters leptin action via reduction in the number of LRb in the plasma membrane. This effect is specific as knockdown of the *BBS1* gene did not alter the trafficking of the endogenous transferrin receptor (in same experiment as Flag-LRb) or the HA-tagged LRa (in separate experiments) (Fig 7B and 7C).

Next, we determined the requirement of other BBSome proteins for LRb trafficking. For this, we tested whether knockdown of the *BBS2* gene, another component of the BBSome, interferes with LRb trafficking to the cell membrane. ShRNA-mediated knockdown of the *BBS2* gene (S10D Fig) reduced surface expression of Flag-LRb, but not HA-LRa or endogenous transferrin receptor (Fig 7D and 7E). Again, this was associated with an attenuated leptin-evoked Stat3 stimulation (S10E Fig). These results demonstrate the importance of the BBSome in mediating LRb trafficking to the cell membrane.

It has been shown that the BBSome mediates cilia membrane protein trafficking through Rab8a, a member of Rab superfamily proteins. We reasoned that Rab8 may also be required for the trafficking of the LRb to the plasma membrane. Cells transfected with a dominant negative form of Rab8a [7] exhibited a significantly reduced Flag-LRb plasma membrane level (Fig 7F and 7G). Interestingly, dominant negative form of Rab8a also reduced the surface levels of HA-LRa and transferrin receptor indicating that Rab8a plays a general role in membrane protein trafficking.

Finally, we tested whether siRNA-mediated knockdown of IFT88 expression alters LRb trafficking to the cell surface levels. However, we found that disruption of IFT88 expression (S10F Fig) had no impact on the membrane levels of Flag-LRb or transferrin receptor (Fig 7H and 7I). These findings demonstrate that IFT88 has no effect on LRb trafficking to the cell membrane. Thus, the role of BBS proteins in mediating LRb trafficking to the cell membrane is specific.

## Discussion

The current study identifies neuronal BBS proteins and the BBSome as key regulators of energy homeostasis through their role in mediating the trafficking of the LRb. We found that mice lacking the *Bbs1* gene in the nervous system develop obesity. Interestingly, *Bbs1* gene ablation from the LRb-expressing cells or in the mediobasal hypothalamus is sufficient to cause to obesity in mice. Moreover, we show that mice lacking the *Bbs1* gene in LRb cells develop obesity as a consequence of both hyperphagia and decreased energy expenditure. On the other hand, ablation of the *Ift88* gene, which is required for ciliogenesis, in LRb-expressing cells caused only marginal weight gain and adiposity. We further show that loss of BBSome subunits, but not IFT88, impairs the trafficking of the LRb to the plasma membrane leading to leptin resistance independently of cilia and obesity. Collectively, these findings demonstrate that obesity in BBS arises from leptin resistance in association with disrupted trafficking of the LRb to the plasma membrane.

Recently, we reported the requirement of BBS proteins for insulin receptor trafficking to the cell membrane and demonstrated that disruption of BBS proteins interfere with insulin receptor surface expression leading to insulin resistance and dysregulated glucose metabolism [37]. Taken together, our findings represent a paradigm shift in the way the role and function of cilia proteins such as BBS are viewed. Indeed, these proteins are commonly thought to be solely associated with ciliary function. However, our studies indicate that BBS proteins are directly involved in the trafficking of receptors to the plasma membrane.

Several mouse models that lack functional *BBS* genes globally display several features seen in BBS patients including obesity [15,18,21]. Our demonstration that the obesity phenotype can largely be reproduced by deleting the *Bbs1* gene either throughout the nervous system, in the mediobasal hypothalamus or selectively in the LRb-expressing cells, but not in adipocytes, highlight the importance of the neurogenic mechanisms for BBS-associated obesity. Interestingly, the sex difference in the development of obesity observed previously in global knockout and knockin mice [15,18–21] was recapitulated in mice lacking the *Bbs1* gene throughout the nervous system, but not when the gene deletion was restricted to the LRb cells suggesting the contribution of cell types other than those expressing the LRb to the sexual dimorphism of obesity in BBS.

The expression of *BBS* genes begin in early development and continue throughout adult life in numerous types of tissue including the nervous system [38–44]. Of note, pre- and post-natal interference with neuronal pathways regulating metabolism can lead to disparate phenotypes [45]. In addition, it has been suggested that obesity in BBS may be caused by neurodevelopmental defects that arises as a consequence of prenatal loss of the *BBS* genes [46]. This is mainly based on the high prevalence of behavioral, neurocognitive and neuroanatomical abnormalities in individuals with BBS, which can manifest in early years [47]. However, our data demonstrate that postnatal deletion of the *Bbs1* gene in the mediobasal hypothalamus can cause obesity in mice, arguing against an early neurodevelopmental origin of obesity in BBS.

Analysis of mice lacking the *Bbs1* gene in the LRb-expressing cells showed that obesity develops as a consequence of both increased food intake and lower energy expenditure. Data also revealed that these mice have reduced leptin sensitivity and LRb signal transduction in a manner independent of obesity. These findings along with the reduced hypothalamic expression of direct targets of the LRb such as *Pomc* gene [4] in *BBS* mice points to leptin resistance as an underlying mechanism of energy imbalance in BBS. This is further supported by our finding that BBS proteins and the BBSome are required for the trafficking of the LRb to the plasma membrane.

In contrast to our findings, however, a recent report has argued that leptin resistance in BBS mice is secondary to obesity [46]. This was based on normal induction of hypothalamic Stat3 activation and anorexia by exogenous leptin in young *Bbs4*^*-/-*^ mice. However, the changes that occur when leptin resistance develops as a secondary consequence of obesity such as in diet-induced obesity are not compatible with what is occurring in BBS. For instance, while increased expression of hypothalamic *Socs3* [32] and *Lrgrp* [34] are hallmark features of diet-induced obesity and key factors in the development of common leptin resistance, the expression of these genes is not altered or tended to be lower in BBS mice. The demonstration that BBS patients exhibit greater circulating leptin levels than weight- and age-matched non-BBS controls [48,49], indicative of pronounced leptin resistance, bolster the notion that dysfunctional leptin signaling is a key pathophysiological mechanism of obesity in BBS.

Cilia-related proteins other than BBS have been implicated in the regulation of energy homeostasis and trafficking of the LRb. Induced ablation of the kinesin-2 subunit KIF3A or IFT88 throughout the nervous system or in the hypothalamus cause obesity in mice [50,51]. Furthermore, the LRb was found to localize in the cilia-basal body in cells and appears required for ciliogenesis [51]. The Retinitis Pigmentosa GTPase Regulator-Interacting Protein-1 Like (*RPGRIP1L*) gene, which encodes a protein localized at the transition zone of the primary cilium was shown to interact physically with the LRb in the vicinity to the cilium upon leptin stimulation [52]. Moreover, mice hypomorphic for *Rpgrip1l* display altered LRb localization and reduced leptin sensitivity and LRb signaling associated with increased body weight and adiposity [52]. These evidences combined with the importance of BBSome for ciliary function, led us to postulate that deleting cilia, through ablation of the *Ift88* gene, from LRb-expressing cells would have the same outcome as loss of *Bbs1* gene. However, loss of cilia in LRb-containing cells caused only a mild increase in body weight and adiposity, which was driven mainly by a reduction in energy expenditure as food intake was not altered. Leptin sensitivity and LRb trafficking were also unaffected by *Ift88* gene ablation. Similarly, mice with induced ablation of the *Ift88* gene in the nervous system exhibited normal responses to leptin when rendered lean by calorie restriction [46]. These findings indicate that processes independent of leptin signaling contribute to the obesity that arises from defects in cilia-related proteins other than BBS [17].

In summary, the present study demonstrates that BBS proteins expressed in the nervous system play a central role in the regulation of energy homeostasis by mediating the trafficking of the LRb. The findings also point to impairment in leptin signaling due to mishandling of the LRb as a major mechanism for energy imbalance leading to obesity in BBS. Another important observation from the current investigation relates to the differential role of the BBS and IFT proteins in the regulation of food intake, energy expenditure and LRb trafficking.

## Materials and Methods

### Ethics statement

Care of the mice used in the experiments met the standards set forth by the National Institutes of Health in their guidelines for the care and use of experimental animals. The study was approved by Institutional Animal Care and Use Committee (IACUC) of the University of Iowa (protocol number 1301003).

### Mice

All animal testing was approved by The University of Iowa Animal Care and Use Committee. Mice expressing the Bbs1^fl/fl^ [23], Ift88^fl/fl^ [36], Nestin^Cre^ [53] and LRb^Cre^ [29] mice were obtained from our colonies. Adip^Cre^ and ROSA (Stop^fl/fl^-tdTomato) reporter transgenic mice were obtained from the Jackson Laboratory. To study the interaction between the LRb and BBS1 protein in the brain, we generated a new transgenic mouse line expressing Flag- and S-tagged human *BBS1* gene under the control of human β–actin promoter. Briefly, human *BBS1* gene was tagged with 3X Flag tag and 2X S tag in its N-terminus and inserted into pSETC-1 vector with human β–actin promoter. The transgene expression was ubiquitous with varying levels in different tissues including the brain. All mice used in this study were on a mixed 129SvEv and C57B/6J backgrounds. Adult mice (12–16 weeks of age) were used unless indicated otherwise.

To specifically delete the *Bbs1* gene from the nervous system or LRb cells we crossed transgenic Nestin^Cre^ or LRb^Cre^ male mice with Bbs1^fl/fl^ female mice. Nestin^Cre^/Bbs1^fl/wt^ and LRb^Cre^/Bbs1^fl/wt^ offspring were subsequently crossed with Bbs1^fl/wt^ mice to generate Nestin^Cre^/Bbs1^fl/fl^ and LRb^Cre^/Bbs1^fl/fl^ mice, respectively. Similar strategy was used to delete the *Bbs1* gene from adipocytes (using the Adip^Cre^ mice) and to selectively delete the *Ift88* gene in the LRb-expressing cells using Ift88^fl/fl^ mice. Presence of the *Cre* transgene, *Bbs1* and *Ift88* alleles, and *td-Tomato* of conditional knockout mice were detected by PCR analysis of tail DNA using the primer sequences provided in S1 Table.

Mice were housed in groups of 3–5 per cage and maintained on 12-h light-dark cycle with lights on at 6 am. Room temperature was maintained at 22°C. Food and water were available *ad libitum* except when the mice were fasted, pair-fed or had their food restricted as described below.

### Adeno-associated viral microinjections

Stereotaxic surgery procedures were performed as previously described [35]. Briefly, 7- to 10-week-old female Bbs1^fl/fl^ mice were anesthetized with i.p. administration of ketamine/xylazine cocktail, and restrained in a Kopf stereotaxic apparatus (Tujunga, CA, USA). A small hole was drilled into the skull under aseptic conditions and a glass micropipette connected to an iontophoresis machine was positioned via the stereotaxic manipulator. Approximately 50 nl of AAV expressing GFP or Cre recombinase was slowly administered into each side of the mediobasal hypothalamic area (-1.50 mm from bregma, ±1.10 mm lateral and -5.90 mm from the surface of the cortex with 8° angle). After bilateral microinjections, the skin incision was closed with surgical staples. Mice were weighed once a week until 25 weeks of age. At the end of study, mice were transcardially perfused with 4% paraformaldehyde and cryopreserved in 20% sucrose. Brains were then sectioned into 40-μm coronal sections and injection sites were examined in every brain (by Zeiss LSM710) using td-Tomato as a read out of Cre-recombinase (“hit” microinjections).

### Body weight and feeding studies

Mice were weighed once a week, beginning at weaning (4 weeks of age) until 25 weeks of age. Total fat mass and lean mass was determined by MRI under anesthesia (91 mg/kg ketamine and 9.1 mg/kg xylazine, i.p.) using a Varian Unity/Inova 4.7 T small-bore MRI system (Varian Inc.). The acquisition consisted of a T1-weighted fast spin-echo sequence (repetition time/echo time = 625/12 ms) with an in-plane resolution of 0.13 x 0.25 mm^2^ and a slice thickness of 1 mm acquired in the axial and coronal planes. In addition, different fat depots and organs were dissected at death and weighed.

To measure food intake, mice were housed individually in regular cages and allowed to acclimate for at least 3 days prior to measurements of 24-hour food intake. In pair-feeding studies, each LRb^Cre^/Bbs1^fl/fl^ animal was given the same amount of food consumed by sex-matched wild-type littermate the day before, beginning at weaning (4 weeks of age) until 15 or 25 weeks of age. Parallel groups of LRb^Cre^/Bbs1^fl/fl^ mice were fed *ad libitum*. In the calorie restriction experiment, each LRb^Cre^/Bbs1^fl/fl^ mouse was given 75–80% of the daily food intake of its control littermate, starting at 4 weeks until 14–16 weeks of age. Normal plasma leptin level of LRb^cre^/Bbs1^fl/fl^ mice relative control littermates was used as an indication of successful normalization of body weight by calorie restriction.

### Effects of leptin on body weight and food intake

Food intake and body weight response to leptin were compared between wild-type and LRb^Cre^/Bbs1^fl/fl^ mice. Baseline body weight and food intake of individually housed mice were measured daily for 5 consecutive days before beginning treatment with vehicle or leptin (1 μg/μl/g body weight i.p., twice per day (8 am and 5 pm) for 4 days).

To test the effect of i.c.v. injection of leptin, a cannula was implanted into the lateral cerebral ventricle as described previously [35]. Mice were allowed at least 1 week to recover from surgery before i.c.v. injection of vehicle (saline, 2 μl) or leptin (2 μg/mouse). Body weight and food intake were recorded 4 and 24 hours after i.c.v. injection.

### Measurements of VO_2_, body temperature and locomotor activity

VO_2_ was measured in mice housed individually in their home cages with free access to food and water as we reported previously [53]. Regular filter lids were replaced with well-sealed filter lids. Room air drawn through each cage was dried by passage through two successive columns of CaSO4 (Drierite, Arcos, Newark, NJ) desiccant, then analyzed for O_2_ (model S-3A/II, AEI Technologies, Pittsburgh, PA) content.

Radiotelemetric measurement was used to record body temperature and locomotor activity in conscious mice using TA10TA-F20 transmitter (Data Sciences, St. Paul, MN) which was implanted in the peritoneal cavity under anesthesia. After 7 days recovery, body temperature and locomotor activity were collected continuously for 6 days and analyzed using Data Science Dataquest software.

### Histology

For histological assessment of fat and liver, mice were perfused with fresh saline, followed by 4% paraformaldehyde (Electron Microscopy Sciences, Hatfield, PA), and peri-gonadal fat pad and liver were dissected, post fixed in 4% paraformaldehyde at 4°C overnight. Tissue blocks were sectioned (10 μm) on a sliding microtome, mounted onto gelatin-coated slides, and stained with standard hematoxylin (peri-gonadal fat) or Oil-Red-O method (liver). Slides were visualized and pictures were obtained using Olympus BX-51 light microscope (ESICO, Davenport, IA). Size of adipocytes was analyzed using National Institutes of Health Image J software (http://rsbweb.nih.gov) as reported previously [53]. At least three slides from each animal were analyzed.

### Analysis of *Bbs1* gene tissue expression

To measure tissue expression of the *Bbs1* gene, total RNA was isolated from 4 different brain regions (hypothalamus, cortex, hippocampus and brainstem), as well as white adipose tissue, liver, kidney and skeletal muscle using RNeasy Plus Mini Kit from Qiagen. Total RNA (1 μg in final volume of 20 μl) was used to synthesize first-strand cDNAs with iScript cDNA Synthesis kit (Bio-Rad). cDNA (5 μl) and primers (0.4 mmol/l) were then added in a final volume of 25 μl PCR mixture (Platinum Supermix, Invitrogen), and amplified in a iQ5 Multicolor Real Time PCR Detection System (Bio-Rad). The PCR conditions for all genes were as follows: denaturation for 5 min at 95°C, 30 cycles for 30 seconds at 95°C, and 30 seconds at 60°C. β–actin mRNA expression was used as an internal control to normalize mRNA expression of these genes. Primer sequences are provided in S1 Table. The sense primer for the *Bbs1* locates to the 3^rd^ exon, which is flunked by two loxP sites, and produces a 536-bp band in Cre-negative mice and no band in Cre-positive mice.

### Hypothalamic gene expression profiling

To analyze expression of various hypothalamic genes, mice were sacrificed by CO_2_ asphyxiation, and total RNA was isolated using RNeasy Plus Mini Kit from Qiagen. Total RNA (5 μg of in final volume of 100 μl) was used to synthesize first-strand cDNAs with the Super-Script pre-amplification system. Then, 10 μl of cDNA and 0.4 mmol/L of primers were added in a final volume of 25 μl PCR mixture (iQ SYBR Green supermix, Bio-Rad), and amplified in an iQ5 Multicolor Real Time PCR Detection System (Bio-Rad). The PCR conditions for all genes were as follow: denaturation for 5 min at 95°C, then 40 cycles for 30 seconds at 95°C and 30 seconds at 60°C. S18 ribosomal RNA expression was used as internal control to normalize mRNA expression of these genes. Primer sequences are provided in S1 Table.

### FACS sorting of LRb-expressing cells and genetic profiling

In order to study the expression of the genes encoding the BBSome and CLP subunits in LRb positive cells, LRb neurons were sorted with flow cytometry. We first established the gating intensities of the tdTomato-positive cells by comparing the fluorescence characteristics of dissociated hypothalamic cells from wild-type mice versus Nestin^Cre^/tdTomato mice (S3A Fig). Briefly, hypothalami from 15 mice were dissected, combined, and frozen in liquid nitrogen. Sliced hypothalami were incubated with 1 ml Acutase (Gibco, A11105-01) at 4°C overnight without shaking, and then passed 10 times through a 0.75 mm diameter glass pipette. Single cells were separated with discontinuous Percoll (Sigma, P1644) density gradient (bottom layer, 3.426 ml Hibernate A, 824.5 μl Percoll and 97.8 μl of 1 M NaCl, middle layer, 3.6 ml Hibernate A, 650.5 μl Percoll and 76.5 μl of 1M NaCl and Top layer, 3.77 ml Hibernate A, 480.3 μl Percoll and 59.5 μl of 1M NaCl) with centrifuge at 2115 rpm for 20 minutes. The pellet containing single cells was resuspended in PBS for flow cytometry. Cell sorting was performed with a Becton Dickinson FACS Aria II (San Jose, CA). Fifty mW of 561 nm light was used to excite td-Tomato while using a 582/15 bandpass filter to collect emissions. Dual parameter plots of forward scatter pulse area (FSC-A) versus side scatter pulse area (SSC-A) and forward scatter pulse area (FSC-A) versus forward scatter pulse width (FSC-W) were used to eliminate dead cells, debris, and aggregates. Twenty thousand gated events were collected first to determine where to set the td-Tomato sort gate hypothalamic cells of LRb^cre^/td-Tomato mice (S3B–S3D Fig). Two hundred fifty thousand LRb (td-Tomato positive) cells were obtained from 18 million cells, and 150 ng of total RNA was isolated using RNeasy Mini Kit from Qiagen. Total RNA in final volume of 100 μl was used to synthesize first-strand cDNAs with the Super-Script pre-amplification system.

To assess the expression of the genes encoding the BBSome and CLP proteins, a two-step amplification technique was used. cDNA (10 μl) and primers (0.4 mmol/l) were added in a final volume of 50 μl PCR mixture (Platinum supermix, Invitrogen), and amplified in a T100 Thermal Cycler PCR Detection System (Bio-Rad). The PCR conditions for all genes were as follows: denaturation for 5 min at 95°C, then 20 cycles for the first round PCR amplification, 30 seconds at 95°C 45 seconds at 65°C and 30 seconds at 72°C. From second round PCR amplification, 10 μl of PCR product from first round PCR application was added in a final volume of 50 μl PCR mixture, denaturation for 5 min at 95°C, 30 cycles for 30 seconds at 95°C, 45 seconds at 65°C and 30 seconds at 72°C. Primer sequences are provided in S1 Table.

### Biochemical assays

To examine the effect of ablating the *Bbs1* gene on the BBSome formation, whole brain and testes were collected from Nestin^Cre^/Bbs1^fl/fl^ mice and control littermates. Brain and testes were homogenized with protein lysate buffer (50 mM HEPES, pH 7.5, 150 mM NaCl, 1 mM MgCl_2_, 1 mM CaCl_2_, 10 mM NaF, 5 mM EDTA, 1% Triton, 2 mM sodium orthovanadate and Roche cocktail protease inhibitor tablet). Interaction between BBS2 and BBS9 was used to detect the BBSome complex by immunoprecipitation. Briefly, BBS2 protein was immunoprecipitated with an anti-BBS2 antibody (Santa Cruz, goat-anti-BBS2, sc-49381(C-16)), the immunocomplex was then separated in a 9% SDS PAGE gel, electrotransferred to a polyvinylidene fluoride membrane, and BBS9 and BBS2 were detected by Western blotting using rabbit antibodies recognizing BBS9 (Sigma, HPA02189, 1:1,000 dilution) or BBS2 (Proteintech Group, 11188-2-AP, 1:1,000 dilution).

To test LRb interaction with BBS1 in the brain of transgenic mice expressing Flag-tagged *BBS1* gene, whole brain was used. Protein samples (800 μg) were subjected to immunoprecipitation assay with 5 μg anti-LR receptor antibody (Santa Cruz, sc-8391), which recognizes both the LRb and LRa. Immunocomplex was separated in 6% SDS PAGE gel, electrotransferred to a polyvinylidene fluoride membrane, then probed with primary antibodies, M2 anti-Flag antibody (Sigma, F1804, 1:1,000), anti-LR antibody (Santa Cruz, sc-8391, 1:1,000) followed by a secondary goat anti-rabbit or goat anti-mouse antibody (1:10,000). Protein expression was visualized with ECL detection kit (GE healthcare).

To detect cell surface receptor expression, cell surface biotinylation assays were performed in HEK 293 cells transiently co-transfected with the pcDNA3-Flag-LRb (Flag-LRb) or pcDNA3-HA-LRa (with a control plasmid on an LKO1) and a plasmid expressing the BBS1-shRNA, BBS2-shRNA or Rab8-DN with X-tremeGENE 9 transfection reagent (Roche). Ift88 siRNA On-Target smartpool (Thermo Scientific) was transfected by Lipofectamine RNAiMAX transfection reagent (Invitrogen). To determine silencing efficacy of BBS1-shRNA and BBS2-shRNA, HA-tagged BBS1 or HA-tagged BBS2 were co-transfected with BBS1-shRNA or BBS2-shRNA plasmid DNA, then their protein expression was examined by Western blotting analysis with HA antibody. For Ift88 siRNA silencing efficacy, real-time RT-PCR was used to measure Ift88 mRNA levels (see primer sequences in S1 Table). Forty eight hours post transfection, the cells were incubated with cleavable EZ-Link Sulfo-NHS-SS-Biotin (Thermo Scientific) for 30 min on ice, and biotinylated proteins were recovered using streptavidin-agarose beads (GE Healthcare). Beads were resuspended in Laemmli buffer, and bound proteins were separated by SDS-PAGE and detected by immunoblotting using mouse M2 anti-Flag (Sigma) to detect Flag-LRb or 3F10 anti-HA (Roche) to detect HA-LRa or mouse anti-transferrin receptor (Invitrogen Inc.) monoclonal antibody. Total Flag-LRb, HA-LRa or transferrin receptor expression in cell lysates was used to normalize cell surface receptors expression.

To measure circulating leptin, plasma was obtained from blood collected from the mice by centrifuging at 2,040g for 10 min. Plasma leptin concentrations were measured by ELISA using a commercially available kit (Bio-Plex Pro Assays, Bio-Rad).

### Western blot analysis

Activation of hypothalamic Stat3 by i.p. or i.c.v. leptin was determined by Western blot analysis. Mice were sacrificed with CO_2_ asphyxiation and the mediobasal hypothalamus was quickly removed. Western blot was also used to examine activation of Stat3 by leptin (10 μM for 15 minutes) in HEK 293 cells transiently transfected by shRNA against *BBS1* or *BBS2* genes.

Proteins were extracted by homogenizing the aorta in tissue lysate buffer (50 mM HEPES, pH 7.5, 150 mM NaCl, 1 mM MgCl_2_, 1 mM CaCl_2_, 10 mM NaF, 5 mM EDTA, 1% Triton, 2 mM sodium orthovanadate and Roche cocktail protease inhibitor tablet). Protein samples (20 μg) were subjected to 9% SDS PAGE gel, electrotransferred on a polyvinylidene fluoride membrane, then probed with primary antibodies against phospho-Stat3 (Cell Signaling, #9131S, 1:1,000) or Stat3 (Cell Signaling, #9132S 1:1,000) followed by a secondary anti-rabbit antibody (1:10,000). Visualization was performed with enhanced chemiluminescence (ECL) detection kit (GE healthcare) followed by autoradiography. Protein expression intensity was analyzed with Image J software.

### Immunofluorescence and confocal microscopy

Mice anesthetized with ketamine and xylazine cocktail were perfused with PBS (5 ml/min; 50 ml) followed by 4% paraformaldehyde/HistoChoice Tissue Fixative (Amresco) in PBS (2.5 ml/min; 50 ml) using Harvard PHD 22/2000 Syringe Pump. Entire brain was excised and incubated in the same fixative overnight at 4°C. Fixed brains were washed 3 times with PBS and incubated in 30% sucrose/PBS overnight with one change of solution after 4–6 hours of initial incubation. Brains were vibratome-sectioned with 40 μm thickness. Immunochemistry was performed on brain sections to detect phosphorylated Stat3 as described previously [35] using a 1:250 dilution of a rabbit polyclonal anti-p-Stat3 antibody (Cell Signaling, #9131S). A cyclase III antibody (Santa Cruz, sc-32113 (N-14), 1:250 dilution) was used to label cilia in the brain. Processed brain sections were mounted using VectaShield mounting medium with DAPI. Images were visualized using confocal microscopy (Zeiss LSM710). Staining of p-Stat3 in the arcuate nucleus was quantified using Image J software.

### Data analysis

Data are expressed as means ± SEM and analyzed using Student’s *t*-test or 1- or 2-way analysis of variance (ANOVA). When ANOVA reached significance, a post-hoc comparison was performed. A P<0.05 was considered statistically significant.

## Supporting Information

S1 Fig Mice lacking the *Bbs1* gene in the nervous system develop obesity. (A—B) Average weekly body weights of female (A) and male (B) Nestin^Cre^/Bbs1^fl/fl^ mice compared to their littermate controls (n = 6–12 per group). (C) Weight of different fat pads of Nestin^Cre^/Bbs1^fl/fl^ mice compared to controls (n = 5 males and 5 females for controls and 5 males and 6 females for Nestin^Cre^/Bbs1^fl/fl^ mice). BAT: brown adipose tissue, RP: retroperitoneal, Ing: inguinal, PG: pero-gonadal. (D—E) Weight of different fat pads in female (D) and male (E) Nestin^Cre^/Bbs1^fl/fl^ mice and littermate controls (n = 5–6 per group). Data are means ± SEM, *P< 0.05 vs control group.
(TIF)

S2 Fig (A—B) Average body weights and weight of different fat pads of Adipo^Cre^/Bbs1^fl/fl^ mice and control littermates (n = 2 males and 3 females for controls and 3 males and 3 females for Adip^Cre^/Bbs1^fl/fl^ mice). (C—D) Representative HE staining (C) and quantification of the mean adipocyte size (D) of peri-gonadal fat pad of 12 weeks old Adipo^cre^/Bbs1^fl/fl^ mice and controls (n = 3 males in each group). Data are means ± SEM.
(TIF)

S3 Fig Validation of FACS to purify brain tdTomato-labelled cells. (A) Comparison of fluorescence characteristics of dissociated cells from hypothalami of wild type mice and Nestin^Cre^/tdTomato mice defines the gating intensities to recognize the tdTomato-positive cells. (B) Sorting of the td-Tomato-positive and -negative hypothalamic cells of LRb^Cre^/tdTomato mice. Arrow indicates the cutoff to collect the tdTomato-positive cells for experiments. (C) Images of dissociated hypothalamic td-Tomato expressing cells of LRb^Cre^/tdTomato mice before and after FACS as viewed through light (left) and fluorescent (right) microscope. Note that before sorting only few cells (arrows) are td-Tomato-positive whereas after sorting nearly 100% of the cells are td-Tomato-positive. (D) Comparison of td-Tomato mRNA expression by PCR between sorted td-Tomato-positive and–negative cells confirming the identity of the FACS-purified cells.
(TIF)

S4 Fig Relevance of *Bbs1* gene in LRb-containing cells for body weight regulation. (A—B) Average weekly body weights of female (A) and male (B) LRb^Cre^/Bbs1^fl/fl^ mice compared to their littermate controls (n = 11–13 per group). (C—D) Weight of different fat pads of 12- (C) and 25-week old (D) LRb^Cre^/Bbs1^fl/fl^ mice and littermate controls (n = 6 males and 5 females for controls and 6 males and 7 females for LRb^Cre^/Bbs1^fl/fl^ mice). (E) Representative confocal images of the hypothalamic arcuate nucleus comparing cilia (ACIII immunostaining) between LRb^Cre^/Bbs1^fl/fl^ and control mice. The arrows point to cilia of LRb-positive cells. Data are means ± SEM, *P< 0.05 vs control group.
(TIF)

S5 Fig Energy imbalance leading to obesity in LRb^cre^/Bbs1^fl/fl^ mice. (A—B) Body weights (A) and fat pad weights (B) of LRb^Cre^/Bbs1^fl/fl^ mice pair-fed from 4 to 15 weeks of age relative to age matched controls and LRb^Cre^/Bbs1^fl/fl^ mice fed *ad libitum* (n = 2 males and 3 females in each group). (C–D) Oxygen consumption (VO_2_, C) and heat generation (D) of Nestin^Cre^/Bbs1^fl/fl^ mice relative to littermate controls (n = 3 males and 3 females in each group). Data are means ± SEM, *P< 0.05 vs. control group.
(TIF)

S6 Fig Leptin resistance in LRb^cre^/Bbs1^fl/fl^ mice. (A–B) Effect of i.p. administration of leptin (1 μg/g bw, twice a day) on food intake (A) and body weight (B) in obese LRb^cre^/Bbs1^fl/fl^ mice and control littermates (n = 4 males and 4 females in each group). (C) Comparison of the relative number of LRb positive cells (labeled with td-Tomato) in the rostral (~Bregma: -1.7 mm), mid (~Bregma: -2.06mm) and caudal (~Bregma: -2.7mm) parts of the arcuate hypothalamic nucleus between LRb^cre^/Bbs1^fl/fl^ mice and control littermates (n = 2 males and 2 females in each group). td-Tomato positive cells were counted manually and expressed as percentage of the controls. (D) mRNA levels of negative regulators of LRb signaling (SOCS3, PTP1B and LRGPR) in the hypothalamus of LRb^cre^/Bbs1^fl/fl^ mice relative to littermate controls (n = 3 males and 3 females in each group). Data are means ± SEM. *P< 0.05 vs control-vehicle and LRb^Cre^/Bbs1^fl/fl^ -leptin groups (A and B) or control group (C), †P< 0.05 vs LRb^Cre^/Bbs1^fl/fl^-vehicle group.
(TIF)

S7 Fig Postnatal deletion of the *Bbs1* gene in the mediobasal hypothalamus increases adiposity. Weight of various fat pads of female Bbs1^fl/fl^ mice that received microinjection of AAV-Cre or AAV-GFP into the mediobasal hypothalamus at 7–10 weeks of age (n = 7 per group). Data are means ± SEM. *P<0.05 vs. AAV-GFP group.
(TIF)

S8 Fig Characterization of mice lacking the *Ift88* gene in LRb cells. (A—B) Body weights of female (A) and male (B) LRb^cre^/Ift88^fl/fl^ mice and littermate controls (n = 11–15 per group). (C—D) Weight of different fat pads of 25 weeks old female (C) and male (D) LRb^cre^/Ift88^fl/fl^ mice and littermate controls (n = 8–9 per group). (E–F) Effect of i.p. administration of vehicle and leptin (1 μg/g bw, twice a day) on food intake (E) and body weight (F) in 6–8 weeks old LRb^cre^/Ift88^fl/fl^ mice and littermate controls (n = 6 per group). Data are means ± SEM. *P< 0.05 vs. control (A-D) or vehicle (E-F).
(TIF)

S9 Fig BBS1 protein interacts with the LRb. (A) Evidence that the anti-LR antibody (Santa Cruz, sc-8391) recognizes both the LRa and LRb in HEK 293 cells transfected with either the pcDNA3-Flag-LRb (Flag-LRb) or pcDNA3-HA-LRa plasmids. (B) Interaction between the Flag-tagged BBS1 and LRb can be detected (based on co-immunoprecipitation assays) on hypothalamic, but not cortex, lysates of transgenic (Tg) mice expressing a Flag-BBS1 protein. IP: immunoprecipitation, IB: immunoblot (these experiments were performed using pooled hypothalami or cortices from 3 mice each).
(TIF)

S10 Fig BBS1 protein regulates LRb trafficking to the cells surface. (A) Efficacy of BBS1-shRNA in HEK 293 cells to reduce HA-tagged BBS1 protein expression. (B—C) Knockdown of BBS1 attenuate leptin-induced activation of Stat3 (B) and S6K (C) in HEK 293 cells. (D) Efficacy of BBS2-shRNA in HEK 293 cells. (E) Knockdown of BBS2 attenuated leptin-induced activation of Stat3 in HEK 293 cells. (F) Efficacy of Ift88-siRNA in HEK 293 cells. Ift88 mRNA expression was measured by real-time RT-PCR (n = 4 per group). Data are means ± SEM. *P< 0.05 vs control group.
(TIF)

S1 Table Primer sequences used. (PDF)

## References

[1] O'Rahilly S (2009) Human genetics illuminates the paths to metabolic disease. Nature 462: 307–314. 10.1038/nature08532 19924209

[2] Zhang Y, Proenca R, Maffei M, Barone M, Leopold L, Friedman JM (1994) Positional cloning of the mouse obese gene and its human homologue. Nature 372: 425–432. 7984236 10.1038/372425a0

[3] Green JS, Parfrey PS, Harnett JD, Farid NR, Cramer BC, Johnson G, Heath O, Mcmanamon PJ, Oleary E, Prysephillips W (1989) The Cardinal Manifestations of Bardet-Biedl Syndrome, A Form of Laurence-Moon-Biedl Syndrome. N Engl J Med 321: 1002–1009. 2779627 10.1056/NEJM198910123211503

[4] Guo DF, Rahmouni K (2011) Molecular basis of the obesity associated with Bardet-Biedl syndrome. Trends Endocrinol Metab 22: 286–293. 10.1016/j.tem.2011.02.009 21514177 PMC3130119

[5] Lindstrand A, Davis EE, Carvalho CM, Pehlivan D, Willer JR, Tsai IC, Ramanathan S, Zuppan C, Sabo A, Muzny D, Gibbs R, Liu P, Lewis RA, Banin E, Lupski JR, Clark R, Katsanis N (2014) Recurrent CNVs and SNVs at the NPHP1 locus contribute pathogenic alleles to Bardet-Biedl syndrome. Am J Hum Genet 94: 745–754. 10.1016/j.ajhg.2014.03.017 24746959 PMC4067552

[6] Zhang Q, Yu D, Seo S, Stone EM, Sheffield VC (2012) Intrinsic protein-protein interaction mediated and chaperonin assisted sequential assembly of a stable Bardet Biedl syndome protein complex, the BBSome. J Biol Chem 287, 20625–35. 10.1074/jbc.M112.341487 22500027 PMC3370246

[7] Nachury MV, Loktev AV, Zhang Q, Westlake CJ, Peranen J, Merdes A, Slusarski DC, Scheller RH, Bazan JF, Sheffield VC, Jackson PK (2007) A core complex of BBS proteins cooperates with the GTPase Rab8 to promote ciliary membrane biogenesis. Cell 129: 1201–1213. 17574030 10.1016/j.cell.2007.03.053

[8] Loktev AV, Zhang QH, Beck JS, Searby CC, Scheetz TE, Bazan JF, Slusarski DC, Sheffield VC, Jackson PK, Nachury MV (2008) A BBSome Subunit Links Ciliogenesis, Microtubule Stability, and Acetylation. Dev Cell 15: 854–865. 10.1016/j.devcel.2008.11.001 19081074

[9] Jin H, White SR, Shida T, Schulz S, Aguiar M, Gygi SP, Bazan JF, Nachury MV (2010) The Conserved Bardet-Biedl Syndrome Proteins Assemble a Coat that Traffics Membrane Proteins to Cilia. Cell 141: 1208–U198. 10.1016/j.cell.2010.05.015 20603001 PMC2898735

[10] Seo S, Baye LM, Schulz NP, Beck JS, Zhang QH, Slusarski DC, Sheffield VC (2010) BBS6, BBS10, and BBS12 form a complex with CCT/TRiC family chaperonins and mediate BBSome assembly. Proc Natl Acad Sci U S A 107: 1488–1493. 10.1073/pnas.0910268107 20080638 PMC2824390

[11] Rosenbaum JL, Witman GB (2002) Intraflagellar transport. Nat Rev Mol Cell Biol 3: 813–825. 12415299 10.1038/nrm952

[12] Kim S, Dynlacht BD (2013) Assembling a primary cilium. Curr Opin Cell Biol 25: 506–511. 10.1016/j.ceb.2013.04.011 23747070 PMC3729615

[13] Li JB, Gerdes JM, Haycraft CJ, Fan YL, Teslovich TM, May-Simera H, Li HT, Blacque OE, Li LY, Leitch CC, Lewis RA, Green JS, Parfrey PS, Leroux MR, Davidson WS, Beales PL, Guay-Woodford LM, Yoder BK, Stormo GD, Katsanis N, Dutcher SK (2004) Comparative and basal genomics identifies a flagellar and basal body proteome that includes the BBS5 human disease gene. Cell 117: 541–552. 15137946 10.1016/s0092-8674(04)00450-7

[14] Chiang AP, Nishimura D, Searby C, Elbedour K, Carmi R, Ferguson AL, Secrist J, Braun T, Casavant T, Stone EM, Sheffield VC (2004) Comparative genomic analysis identifies an ADP-ribosylation factor-like gene as the cause of Bardet-Biedl syndrome (BBS3). Am J Hum Genet 75: 475–484. 15258860 10.1086/423903 PMC1182025

[15] Davis RE, Swiderski RE, Rahmouni K, Nishimura DY, Mullins RF, Agassandian K, Philp AR, Searby CC, Andrews MP, Thompson S, Berry CJ, Thedens DR, Yang B, Weiss RM, Cassell MD, Stone EM, Sheffield VC (2007) A knockin mouse model of the Bardet-Biedl syndrome 1 M390R mutation has cilia defects, ventriculomegaly, retinopathy, and obesity. Proc Natl Acad Sci U S A 104: 19422–19427. 18032602 10.1073/pnas.0708571104 PMC2148305

[16] Badano JL, Mitsuma N, Beales PL, Katsanis N (2006) The ciliopathies: An emerging class of human genetic disorders. Ann Rev Gen Hum Genet 7: 125–148. 10.1146/annurev.genom.7.080505.115610 16722803

[17] Quinlan RJ, Tobin JL, Beales PL (2008) Modeling ciliopathies: Primary cilia in development and disease. Curr Top Dev Biol 84: 249–310. 10.1016/S0070-2153(08)00605-4 19186246

[18] Eichers ER, Abd-El-Barr MM, Paylor R, Lewis RA, Bi WM, Lin XD, Meehan TP, Stockton DW, Wu SM, Lindsay E, Justice MJ, Beales PL, Katsanis N, Lupski JR (2006) Phenotypic characterization of Bbs4 null mice reveals age-dependent penetrance and variable expressivity. Hum Genet 120: 211–226. 16794820 10.1007/s00439-006-0197-y

[19] Fath MA, Mullins RF, Searby C, Nishimura DY, Wei J, Rahmouni K, Davis RE, Tayeh MK, Andrews M, Yang B, Sigmund CD, Stone EM, Sheffield VC (2005) Mkks-null mice have a phenotype resembling Bardet-Biedl syndrome. Hum Mol Genet 14: 1109–1118. 15772095 10.1093/hmg/ddi123

[20] Nishimura DY, Fath M, Mullins RF, Searby C, Andrews M, Davis R, Andorf JL, Mykytyn K, Swiderski RE, Yang BL, Carmi R, Stone EM, Sheffield VC (2004) Bbs2-null mice have neurosensory deficits, a defect in social dominance, and retinopathy associated with mislocalization of rhodopsin. Proc Natl Acad Sci U S A 101: 16588–16593. 15539463 10.1073/pnas.0405496101 PMC534519

[21] Rahmouni K, Fath MA, Seo S, Thedens DR, Berry CJ, Weiss R, Nishimura DY, Sheffield VC (2008) Leptin resistance contributes to obesity and hypertension in mouse models of Bardet-Biedl syndrome. J Clin Invest 118: 1458–1467. 10.1172/JCI32357 18317593 PMC2262028

[22] Seo SJ, Guo DF, Bugge K, Morgan DA, Rahmouni K, Sheffield VC (2009) Requirement of Bardet-Biedl syndrome proteins for leptin receptor signaling. Hum Mol Genet 18: 1323–1331. 10.1093/hmg/ddp031 19150989 PMC2655773

[23] Carter CS, Vogel TW, Zhang Q, Seo S, Swiderski RE, Moninger TO, Cassell MD, Thedens DR, Keppler-Noreuil KM, Nopoulos P, Nishimura DY, Searby CC, Bugge K, Sheffield VC (2012) Abnormal development of NG2+PDGFR-alpha+ neural progenitor cells leads to neonatal hydrocephalus in a ciliopathy mouse model. Nat Med 18: 1797–1804. n 10.1038/nm.2996 23160237 PMC3684048

[24] Marion V, Stoetzel C, Schlicht D, Messaddeq N, Koch M, Flori E, Danse JM, Mandel JL, Dollfus H (2009) Transient ciliogenesis involving Bardet- Biedl syndrome proteins is a fundamental characteristic of adipogenic differentiation. Proc Natl Acad Sci U S A 106: 1820–1825. 10.1073/pnas.0812518106 19190184 PMC2635307

[25] Aksanov O, Green P, Birk RZ (2014) BBS4 directly affects proliferation and differentiation of adipocytes. Cell Mol Life Sci 71(17):3381–92. 10.1007/s00018-014-1571-x 24500759 PMC11113930

[26] Wang ZV, Deng Y, Wang QA, Sun K, Scherer PE (2010) Identification and characterization of a promoter cassette conferring adipocyte-specific gene expression. Endocrinology 151: 2933–2939. 10.1210/en.2010-0136 20363877 PMC2875825

[27] Friedman JM, Mantzoros CS (2015) 20 years of leptin: from the discovery of the leptin gene to leptin in our therapeutic armamentarium. Metabolism 64: 1–4. 10.1016/j.metabol.2014.10.023 25497341

[28] DeFalco J, Tomishima M, Liu HY, Zhao C, Cai XL, Marth JD, Enquist L, Friedman JM (2001) Virus-assisted mapping of neural inputs to a feeding center in the hypothalamus. Science 291: 2608–2613. 11283374 10.1126/science.1056602

[29] Plum L, Rother E, Munzberg H, Wunderlich FT, Morgan DA, Hampel B, Shanabrough M, Janoschek R, Konner AC, Alber J, Suzuki A, Krone W, Horvath TL, Rahmouni K, Bruning JC (2007) Enhanced leptin-stimulated Pi3k activation in the CNS promotes white adipose tissue transdifferentiation. Cell Metab 6: 431–445. 18054313 10.1016/j.cmet.2007.10.012

[30] Hempel CM, Sugino K, Nelson SB (2007) A manual method for the purification of fluorescently labeled neurons from the mammalian brain. Nat Protoc 2: 2924–2929. 18007629 10.1038/nprot.2007.416

[31] Saxena A, Wagatsuma A, Noro Y, Kuji T, Asaka-Oba A, Watahiki A, Gurnot C, Fagiolini M, Hensch TK, Carninci P (2012) Trehalose-enhanced isolation of neuronal sub-types from adult mouse brain. Biotechniques 52: 381–385. 10.2144/0000113878 22668417 PMC3696583

[32] Bjorbaek C, Elmquist JK, Frantz JD, Shoelson SE, Flier JS (1998) Identification of SOCS-3 as a potential mediator of central leptin resistance. Mol Cell 1: 619–625. 9660946 10.1016/s1097-2765(00)80062-3

[33] Zabolotny JM, Bence-Hanulec KK, Stricker-Krongrad A, Haj F, Wang Y, Minokoshi Y, Kim YB, Elmquist JK, Tartaglia LA, Kahn BB, Neel BG (2002) PTP1B regulates leptin signal transduction in vivo. Dev Cell 2: 489–495. 11970898 10.1016/s1534-5807(02)00148-x

[34] Couturier C, Sarkis C, Seron K, Belouzard S, Chen P, Lenain A, Corset L, Dam J, Vauthier V, Dubartt A, Mallet J, Froguel P, Rouille Y, Jockers R (2007) Silencing of OB-RGRP in mouse hypothalamic arcuate nucleus increases leptin receptor signaling and prevents diet-induced obesity. Proc Natl Acad Sci U S A 104: 19476–19481. 18042720 10.1073/pnas.0706671104 PMC2148314

[35] Harlan SM, Morgan DA, Agassandian K, Guo DF, Cassell MD, Sigmund CD, Mark AL, Rahmouni K (2011) Ablation of the Leptin Receptor in the Hypothalamic Arcuate Nucleus Abrogates Leptin-Induced Sympathetic Activation. Circ Res 108: 808–812. 10.1161/CIRCRESAHA.111.240226 21311043 PMC3072835

[36] Haycraft CJ, Zhang Q, Song B, Jackson WS, Detloff PJ, Serra R, Yoder BK (2007) Intraflagellar transport is essential for endochondral bone formation. Development 134: 307–316. 17166921 10.1242/dev.02732

[37] Starks RD, Beyer AM, Guo DF, Boland L, Zhang Q, Sheffield VC, Rahmouni K (2015) Regulation of Insulin Receptor Trafficking by Bardet Biedl Syndrome Proteins. PLoS Genet 11: e1005311 10.1371/journal.pgen.1005311 26103456 PMC4478011

[38] Fan Y, Esmail MA, Ansley SJ, Blacque OE, Boroevich K, Ross AJ, Moore SJ, Badano JL, May-Simera H, Compton DS, Green JS, Lewis RA, van Haelst MM, Parfrey PS, Baillie DL, Beales PL, Katsanis N, Davidson WS, Leroux MR (2004) Mutations in a member of the Ras superfamily of small GTP-binding proteins causes Bardet-Biedl syndrome. Nat Genet 36: 989–93. 15314642 10.1038/ng1414

[39] Katsanis N, Beales PL, Woods MO, Lewis RA, Green JS, Parfrey PS, Ansley SJ, Davidson WS, Lupski JR (2000) Mutations in MKKS cause obesity, retinal dystrophy and renal malformations associated with bardet-biedl syndrome. Nat Genet 26: 67–70. 10973251 10.1038/79201

[40] Kim JC, Badano JL, Sibold S, Esmail MA, Hill J, Hoskins BE, Leitch CC, Venner K, Ansley SJ, Ross AJ, Leroux MR, Katsanis N, Beales PL (2004) The Bardet-Biedl protein BBS4 targets cargo to the pericentriolar region and is required for microtubule anchoring and cell cycle progression. Nat Genet 36: 462–70. 15107855 10.1038/ng1352

[41] Mykytyn K, Mullins RF, Andrews M, Chiang AP, Swiderski RE, Yang B, Braun T, Casavant T, Stone EM, Sheffield VC (2004) Bardet-Biedl syndrome type 4 (BBS4)-null mice implicate Bbs4 in flagella formation but not global cilia assembly. Proc Natl Acad Sci U S A 101: 8664–9. 15173597 10.1073/pnas.0402354101 PMC423252

[42] Nishimura DY, Searby CC, Carmi R, Elbedour K, Van Maldergem L, Fulton AB, Lam BL, Powell BR, Swiderski RE, Bugge KE, Haider NB, Kwitek-Black AE, Ying L, Duhl DM, Gorman SW, Heon E, Iannaccone A, Bonneau D, Biesecker LG, Jacobson SG, Stone EM, Sheffield VC (2001) Positional cloning of a novel gene on chromosome 16q causing Bardet-Biedl syndrome (BBS2). Hum Mol Genet 10: 865–74. 11285252 10.1093/hmg/10.8.865

[43] Pasqualato S, Renault L, Cherfils J (2002) Arf, Arl, Arp and Sar proteins: a family of GTP-binding proteins with a structural device for 'front-back' communication. EMBO Rep 3: 1035–41. 12429613 10.1093/embo-reports/kvf221 PMC1307594

[44] Slavotinek AM, Stone EM, Mykytyn K, Heckenlively JR, Green JS, Heon E, Musarella MA, Parfrey PS, Sheffield VC, Biesecker LG (2000) Mutations in MKKS cause bardet-biedl syndrome. Nat Genet 26: 15–6. 10973238 10.1038/79116

[45] Luquet S, Perez FA, Hnasko TS, Palmiter RD (2005) NPY/AgRP neurons are essential for feeding in adult mice but can be ablated in neonates. Science 310: 683–685. 16254186 10.1126/science.1115524

[46] Berbari NF, Pasek RC, Malarkey EB, Yazdi SM, McNair AD, Lewis WR, Nagy TR, Kesterson RA, Yoder BK (2013) Leptin resistance is a secondary consequence of the obesity in ciliopathy mutant mice. Proc Natl Acad Sci U S A 110: 7796–7801. 10.1073/pnas.1210192110 23599282 PMC3651481

[47] Brinckman DD, Keppler-Noreuil KM, Blumhorst C, Biesecker LG, Sapp JC, Johnston JJ, Wiggs EA (2013) Cognitive, sensory, and psychosocial characteristics in patients with Bardet-Biedl syndrome. Am J Med Genet A 161A: 2964–2971. 10.1002/ajmg.a.36245 24194441 PMC4419571

[48] Feuillan PP, Ng D, Han JC, Sapp JC, Wetsch K, Spaulding E, Zheng YQC, Caruso RC, Brooks BP, Johnston JJ, Yanovski JA, Biesecker LG (2011) Patients with Bardet-Biedl Syndrome Have Hyperleptinemia Suggestive of Leptin Resistance. J Clin Endocrinol Metab 96: E528–E535. 10.1210/jc.2010-2290 21209035 PMC3047221

[49] Marion V, Mockel A, De MC, Obringer C, Claussmann A, Simon A, Messaddeq N, Durand M, Dupuis L, Loeffler JP, King P, Mutter-Schmidt C, Petrovsky N, Stoetzel C, Dollfus H (2012) BBS-induced ciliary defect enhances adipogenesis, causing paradoxical higher-insulin sensitivity, glucose usage, and decreased inflammatory response. Cell Metab 16: 363–377. 10.1016/j.cmet.2012.08.005 22958920

[50] Davenport JR, Watts AJ, Roper VC, Croyle MJ, van Groen T, Wyss JM, Nagy TR, Kesterson RA, Yoder BK (2007) Disruption of intraflagellar in adult mice leads to transport obesity and slow-onset cystic kidney disease. Curr Biol 17: 1586–1594. 17825558 10.1016/j.cub.2007.08.034 PMC2084209

[51] Han YM, Kang GM, Byun K, Ko HW, Kim J, Shin MS, Kim HK, Gil SY, Yu JH, Lee B, Kim MS (2014) Leptin-promoted cilia assembly is critical for normal energy balance. J Clin Invest 124: 2193–2197. 10.1172/JCI69395 24667636 PMC4001529

[52] Stratigopoulos G, Martin Carli JF, O'Day DR, Wang L, Leduc CA, Lanzano P, Chung WK, Rosenbaum M, Egli D, Doherty DA, Leibel RL (2014) Hypomorphism for RPGRIP1L, a ciliary gene vicinal to the FTO locus, causes increased adiposity in mice. Cell Metab 19: 767–779. 10.1016/j.cmet.2014.04.009 24807221 PMC4131684

[53] Zhang ZM, Liu XB, Morgan DA, Kuburas A, Thedens DR, Russo AF, Rahmouni K (2011) Neuronal Receptor Activity-Modifying Protein 1 Promotes Energy Expenditure in Mice. Diabetes 60: 1063–1071. 10.2337/db10-0692 21357463 PMC3064080

