# Supplementary figures and images for: The BBSome Controls Energy Homeostasis by Mediating the Transport of the Leptin Receptor to the Plasma Membrane
Source: PLoS Genet. 2016 Feb 29;12(2):e1005890. doi: 10.1371/journal.pgen.1005890 (PMC4771807; doi:10.1371/journal.pgen.1005890)

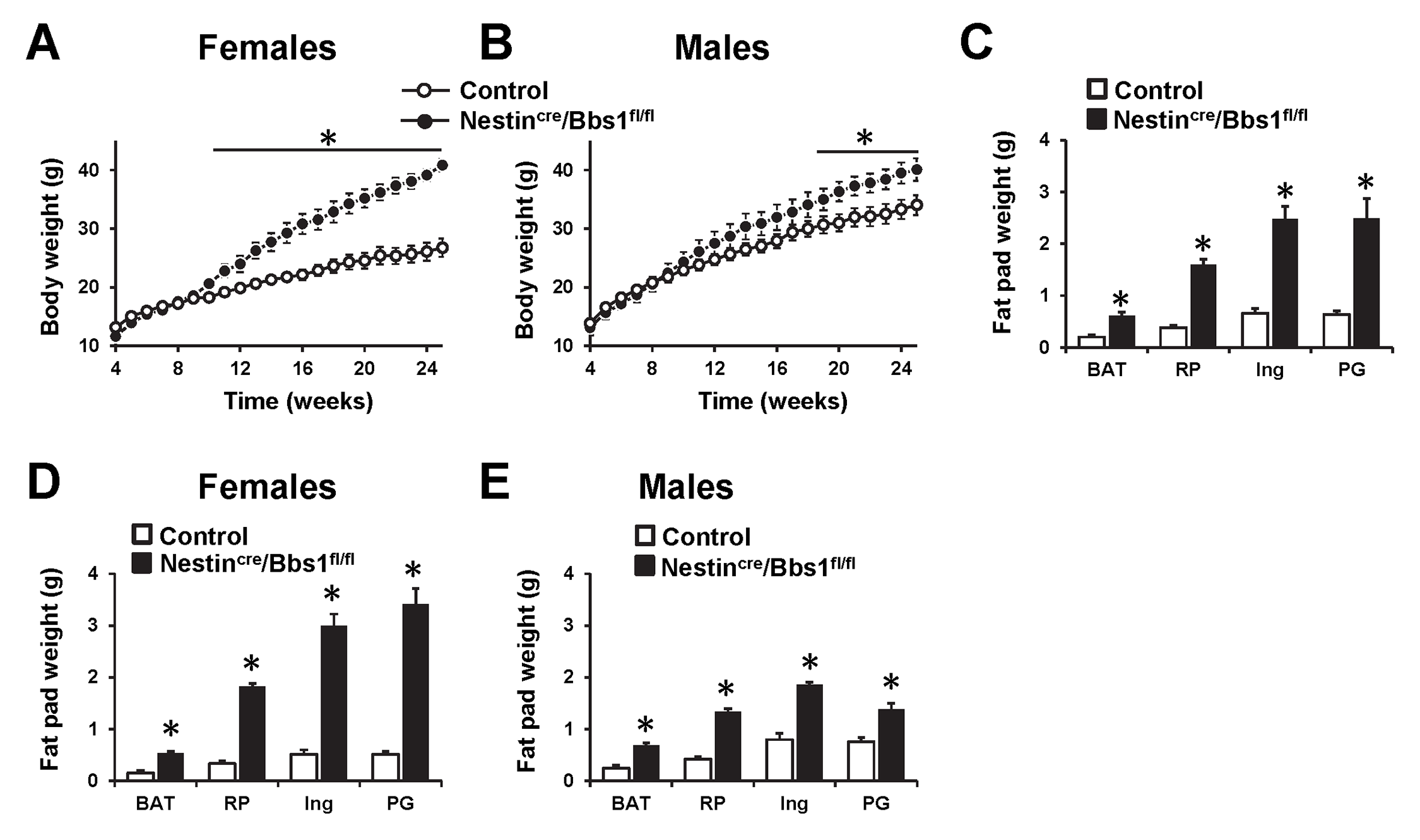

Supplement: S1 Fig — (A—B) Average weekly body weights of female (A) and male (B) NestinCre/Bbs1fl/fl mice compared to their littermate controls (n = 6–12 per group). (C) Weight of different fat pads of NestinCre/Bbs1fl/fl mice compared to controls (n = 5 males and 5 females for controls and 5 males and 6 females for NestinCre/Bbs1fl/fl mice). BAT: brown adipose tissue, RP: retroperitoneal, Ing: inguinal, PG: pero-gonadal. (D—E) Weight of different fat pads in female (D) and male (E) NestinCre/Bbs1fl/fl mice and littermate controls (n = 5–6 per group). Data are means ± SEM, *P< 0.05 vs control group. (TIF) [file pgen.1005890.s001.tif]

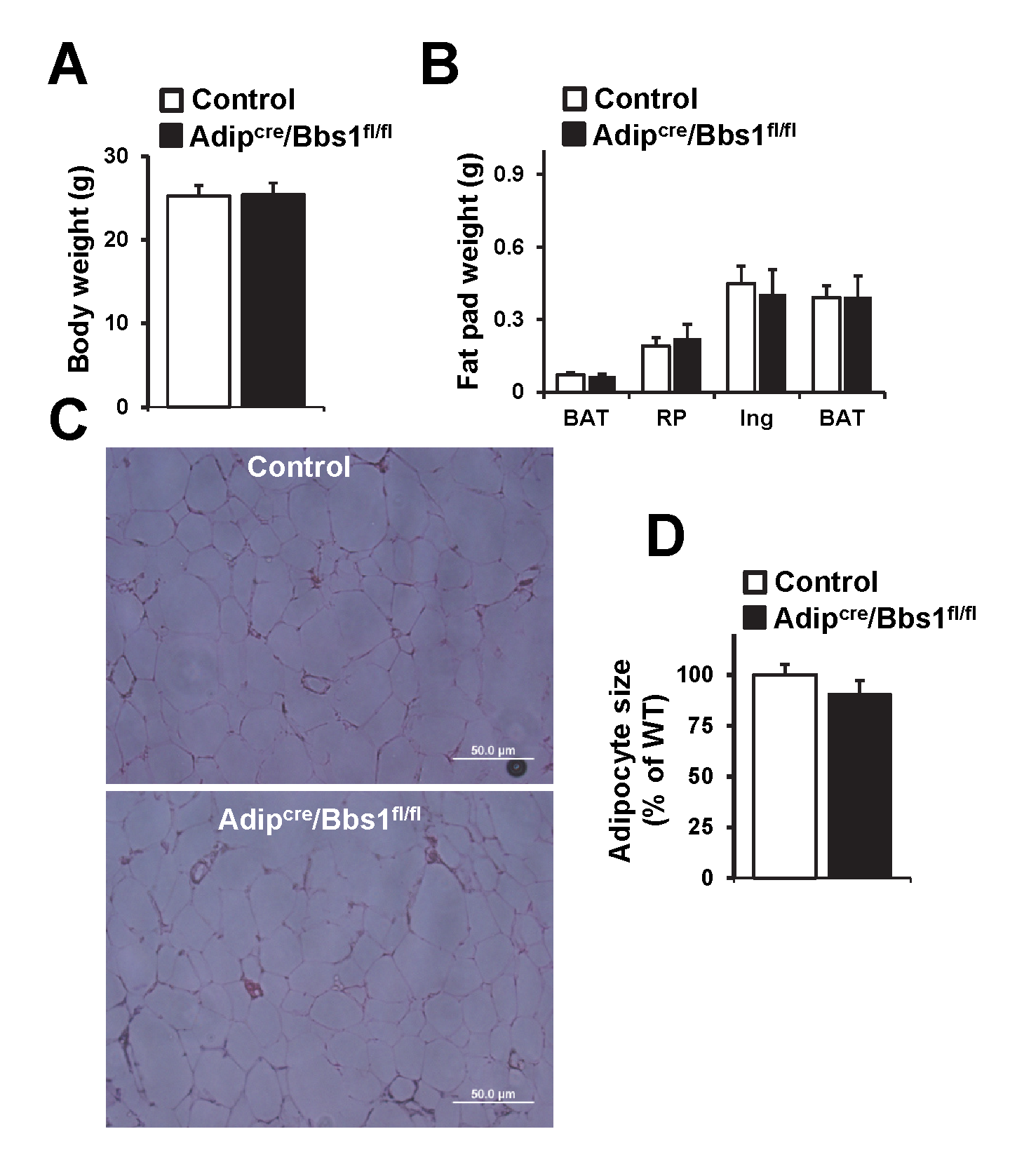

Supplement: S2 Fig — (A—B) Average body weights and weight of different fat pads of AdipoCre/Bbs1fl/fl mice and control littermates (n = 2 males and 3 females for controls and 3 males and 3 females for AdipCre/Bbs1fl/fl mice). (C—D) Representative HE staining (C) and quantification of the mean adipocyte size (D) of peri-gonadal fat pad of 12 weeks old Adipocre/Bbs1fl/fl mice and controls (n = 3 males in each group). Data are means ± SEM. (TIF) [file pgen.1005890.s002.tif]

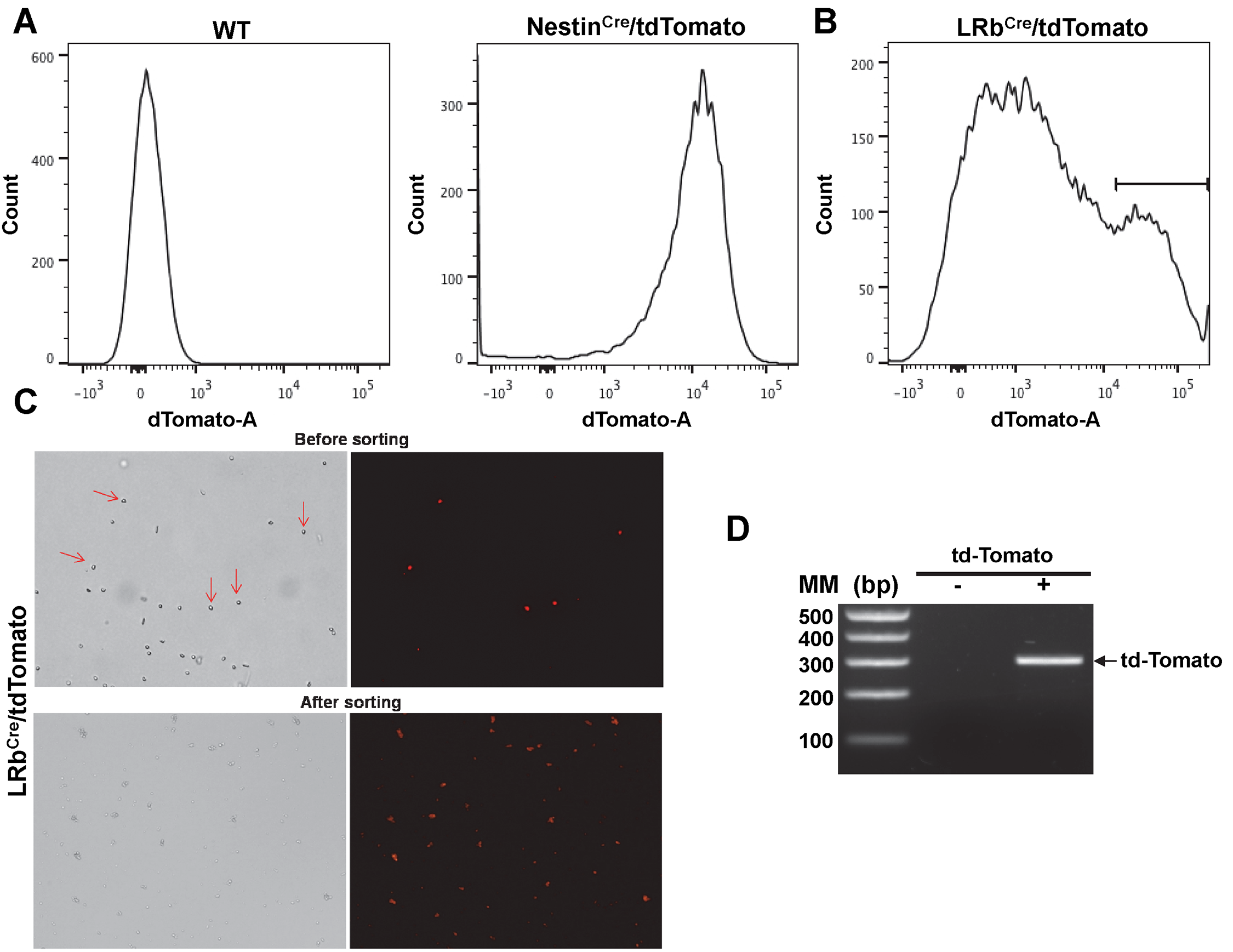

Supplement: S3 Fig — (A) Comparison of fluorescence characteristics of dissociated cells from hypothalami of wild type mice and NestinCre/tdTomato mice defines the gating intensities to recognize the tdTomato-positive cells. (B) Sorting of the td-Tomato-positive and -negative hypothalamic cells of LRbCre/tdTomato mice. Arrow indicates the cutoff to collect the tdTomato-positive cells for experiments. (C) Images of dissociated hypothalamic td-Tomato expressing cells of LRbCre/tdTomato mice before and after FACS as viewed through light (left) and fluorescent (right) microscope. Note that before sorting only few cells (arrows) are td-Tomato-positive whereas after sorting nearly 100% of the cells are td-Tomato-positive. (D) Comparison of td-Tomato mRNA expression by PCR between sorted td-Tomato-positive and–negative cells confirming the identity of the FACS-purified cells. (TIF) [file pgen.1005890.s003.tif]

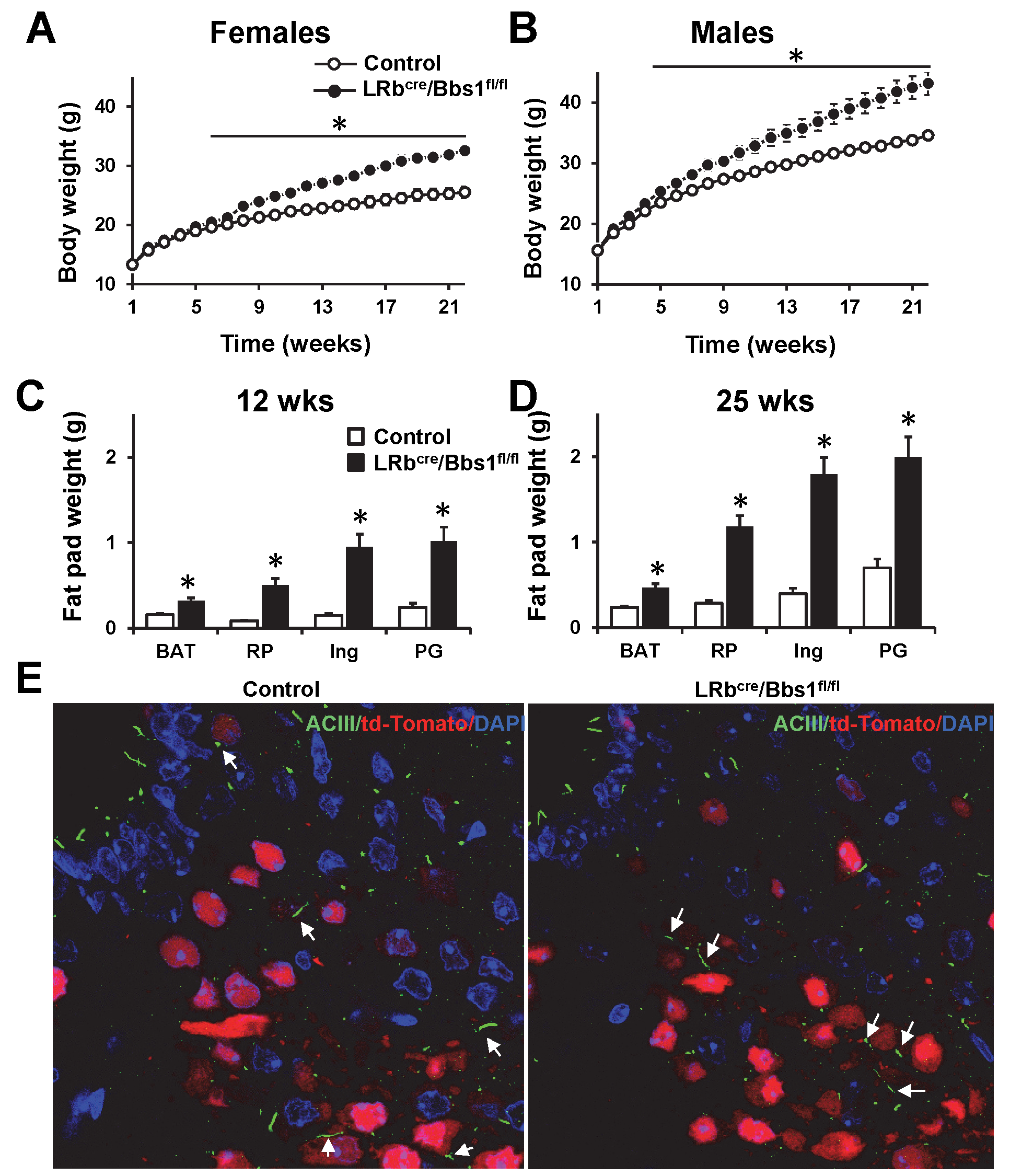

Supplement: S4 Fig — (A—B) Average weekly body weights of female (A) and male (B) LRbCre/Bbs1fl/fl mice compared to their littermate controls (n = 11–13 per group). (C—D) Weight of different fat pads of 12- (C) and 25-week old (D) LRbCre/Bbs1fl/fl mice and littermate controls (n = 6 males and 5 females for controls and 6 males and 7 females for LRbCre/Bbs1fl/fl mice). (E) Representative confocal images of the hypothalamic arcuate nucleus comparing cilia (ACIII immunostaining) between LRbCre/Bbs1fl/fl and control mice. The arrows point to cilia of LRb-positive cells. Data are means ± SEM, *P< 0.05 vs control group. (TIF) [file pgen.1005890.s004.tif]

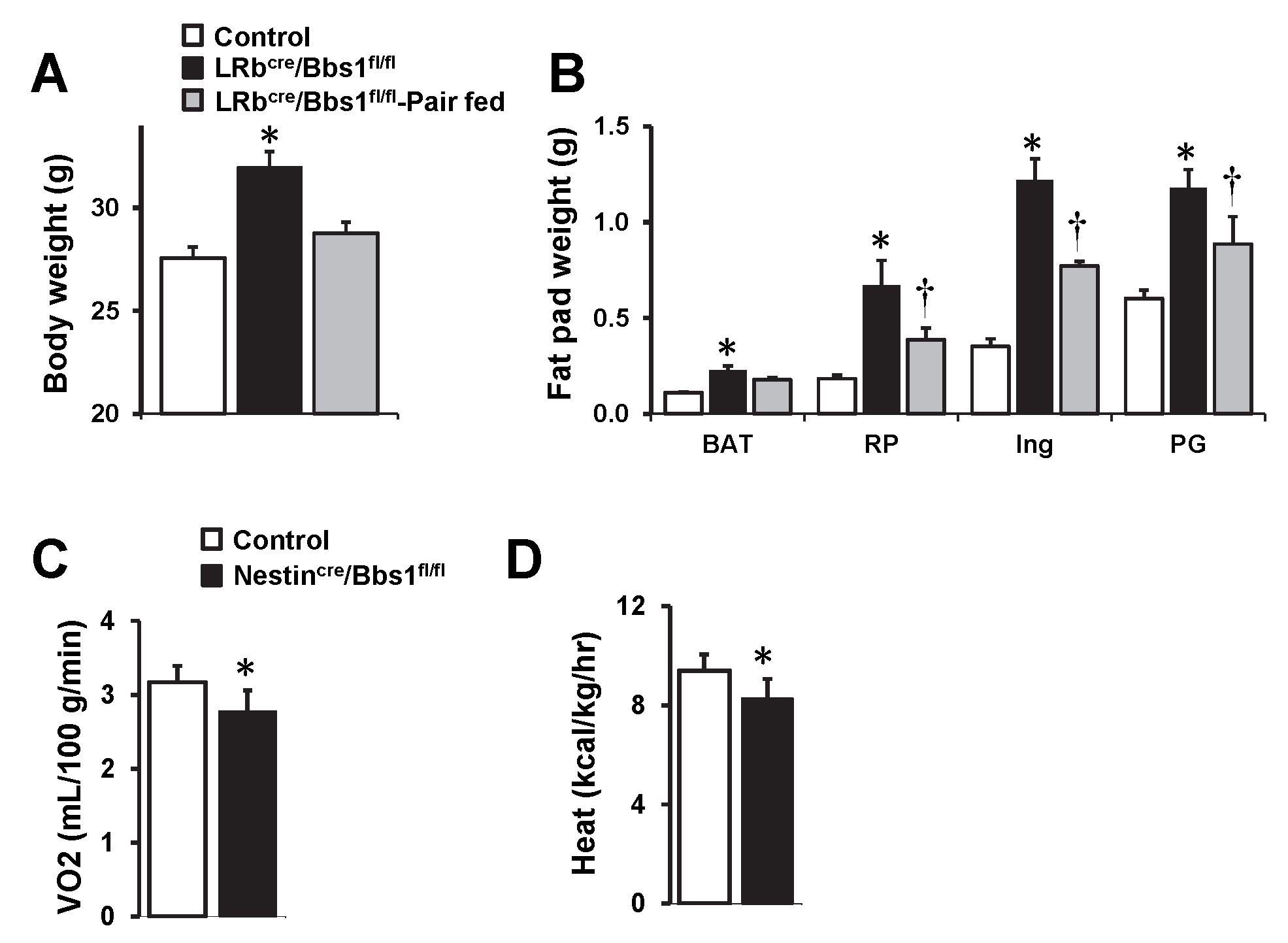

Supplement: S5 Fig — (A—B) Body weights (A) and fat pad weights (B) of LRbCre/Bbs1fl/fl mice pair-fed from 4 to 15 weeks of age relative to age matched controls and LRbCre/Bbs1fl/fl mice fed ad libitum (n = 2 males and 3 females in each group). (C–D) Oxygen consumption (VO2, C) and heat generation (D) of NestinCre/Bbs1fl/fl mice relative to littermate controls (n = 3 males and 3 females in each group). Data are means ± SEM, *P< 0.05 vs. control group. (TIF) [file pgen.1005890.s005.tif]

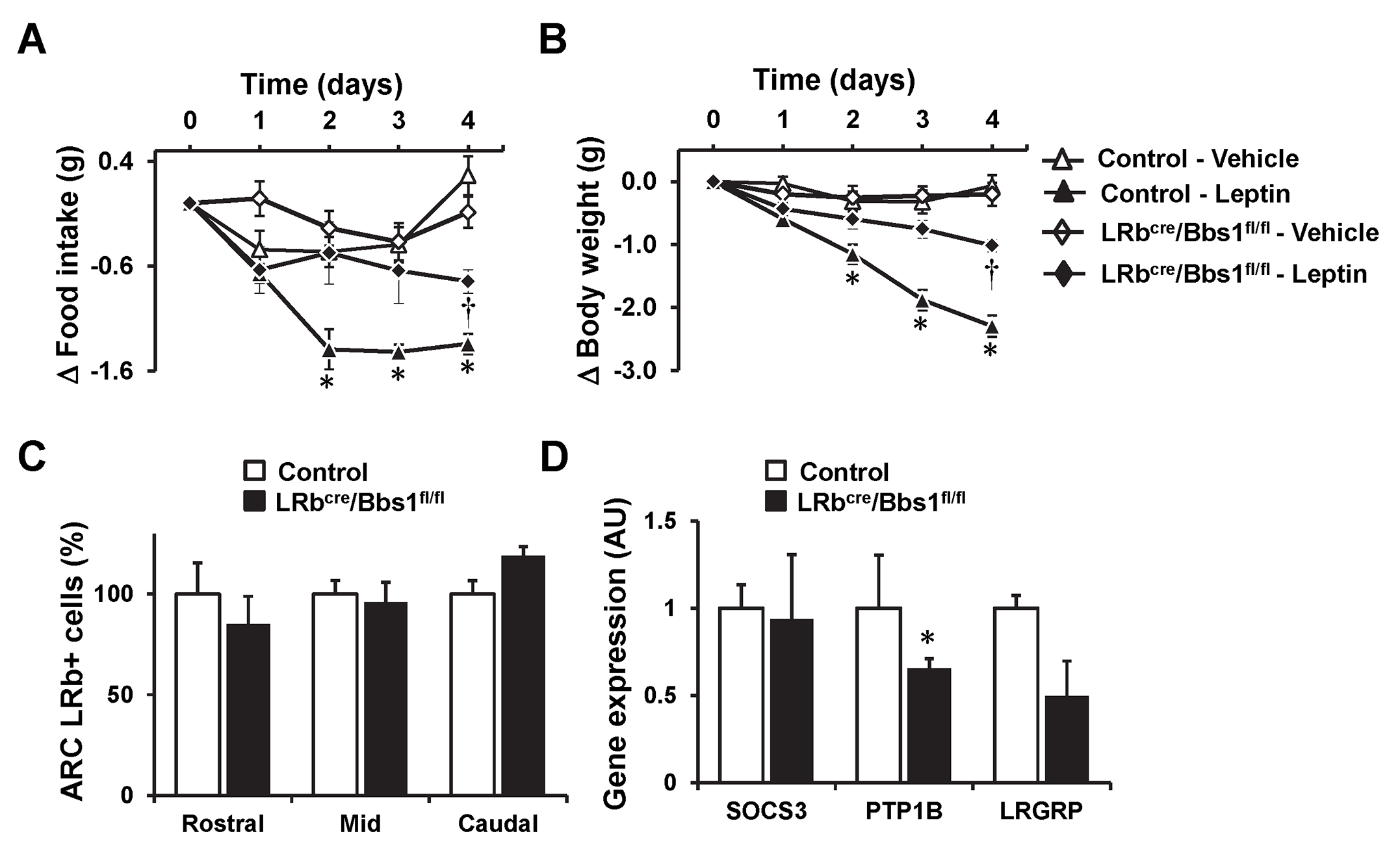

Supplement: S6 Fig — (A–B) Effect of i.p. administration of leptin (1 μg/g bw, twice a day) on food intake (A) and body weight (B) in obese LRbcre/Bbs1fl/fl mice and control littermates (n = 4 males and 4 females in each group). (C) Comparison of the relative number of LRb positive cells (labeled with td-Tomato) in the rostral (~Bregma: -1.7 mm), mid (~Bregma: -2.06mm) and caudal (~Bregma: -2.7mm) parts of the arcuate hypothalamic nucleus between LRbcre/Bbs1fl/fl mice and control littermates (n = 2 males and 2 females in each group). td-Tomato positive cells were counted manually and expressed as percentage of the controls. (D) mRNA levels of negative regulators of LRb signaling (SOCS3, PTP1B and LRGPR) in the hypothalamus of LRbcre/Bbs1fl/fl mice relative to littermate controls (n = 3 males and 3 females in each group). Data are means ± SEM. *P< 0.05 vs control-vehicle and LRbCre/Bbs1fl/fl -leptin groups (A and B) or control group (C), †P< 0.05 vs LRbCre/Bbs1fl/fl-vehicle group. (TIF) [file pgen.1005890.s006.tif]

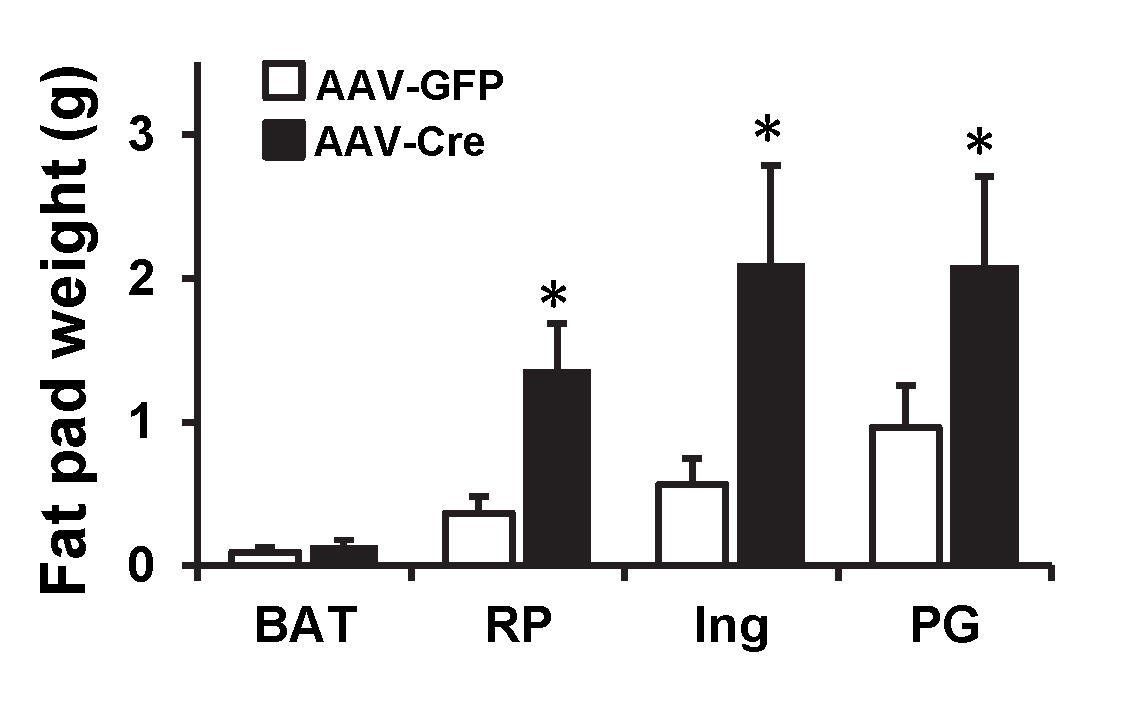

Supplement: S7 Fig — Weight of various fat pads of female Bbs1fl/fl mice that received microinjection of AAV-Cre or AAV-GFP into the mediobasal hypothalamus at 7–10 weeks of age (n = 7 per group). Data are means ± SEM. *P<0.05 vs. AAV-GFP group. (TIF) [file pgen.1005890.s007.tif]

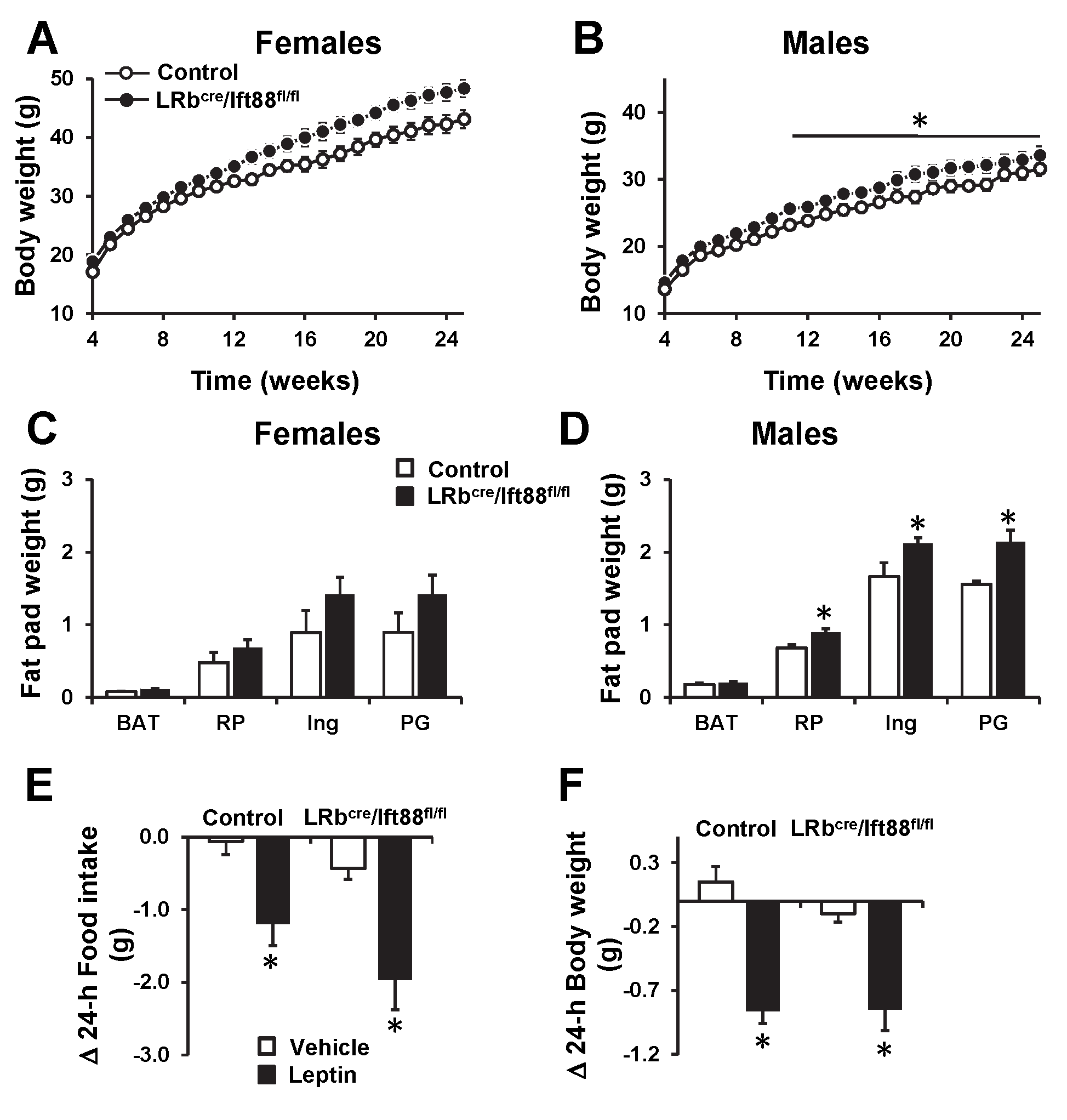

Supplement: S8 Fig — (A—B) Body weights of female (A) and male (B) LRbcre/Ift88fl/fl mice and littermate controls (n = 11–15 per group). (C—D) Weight of different fat pads of 25 weeks old female (C) and male (D) LRbcre/Ift88fl/fl mice and littermate controls (n = 8–9 per group). (E–F) Effect of i.p. administration of vehicle and leptin (1 μg/g bw, twice a day) on food intake (E) and body weight (F) in 6–8 weeks old LRbcre/Ift88fl/fl mice and littermate controls (n = 6 per group). Data are means ± SEM. *P< 0.05 vs. control (A-D) or vehicle (E-F). (TIF) [file pgen.1005890.s008.tif]

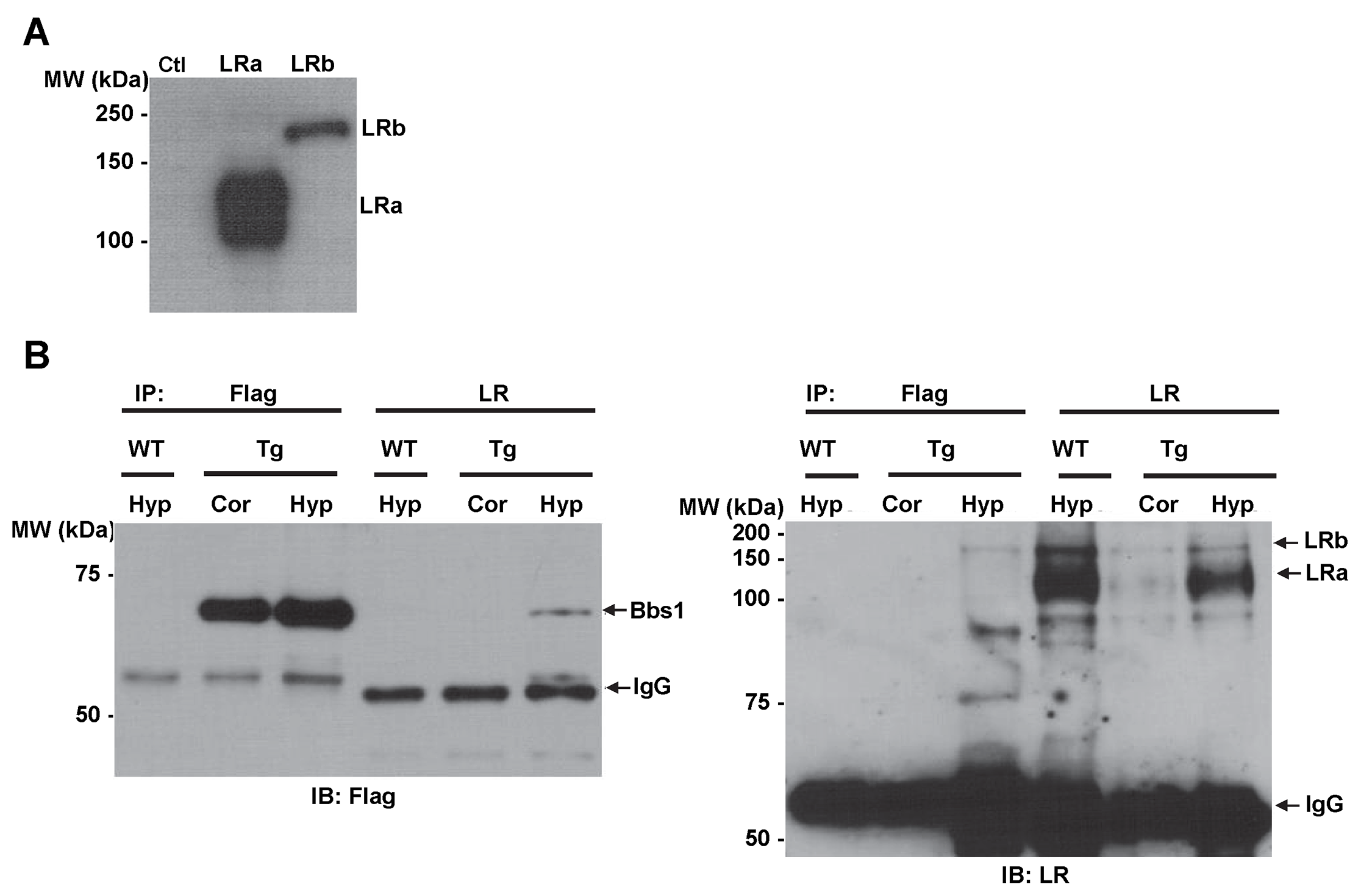

Supplement: S9 Fig — (A) Evidence that the anti-LR antibody (Santa Cruz, sc-8391) recognizes both the LRa and LRb in HEK 293 cells transfected with either the pcDNA3-Flag-LRb (Flag-LRb) or pcDNA3-HA-LRa plasmids. (B) Interaction between the Flag-tagged BBS1 and LRb can be detected (based on co-immunoprecipitation assays) on hypothalamic, but not cortex, lysates of transgenic (Tg) mice expressing a Flag-BBS1 protein. IP: immunoprecipitation, IB: immunoblot (these experiments were performed using pooled hypothalami or cortices from 3 mice each). (TIF) [file pgen.1005890.s009.tif]

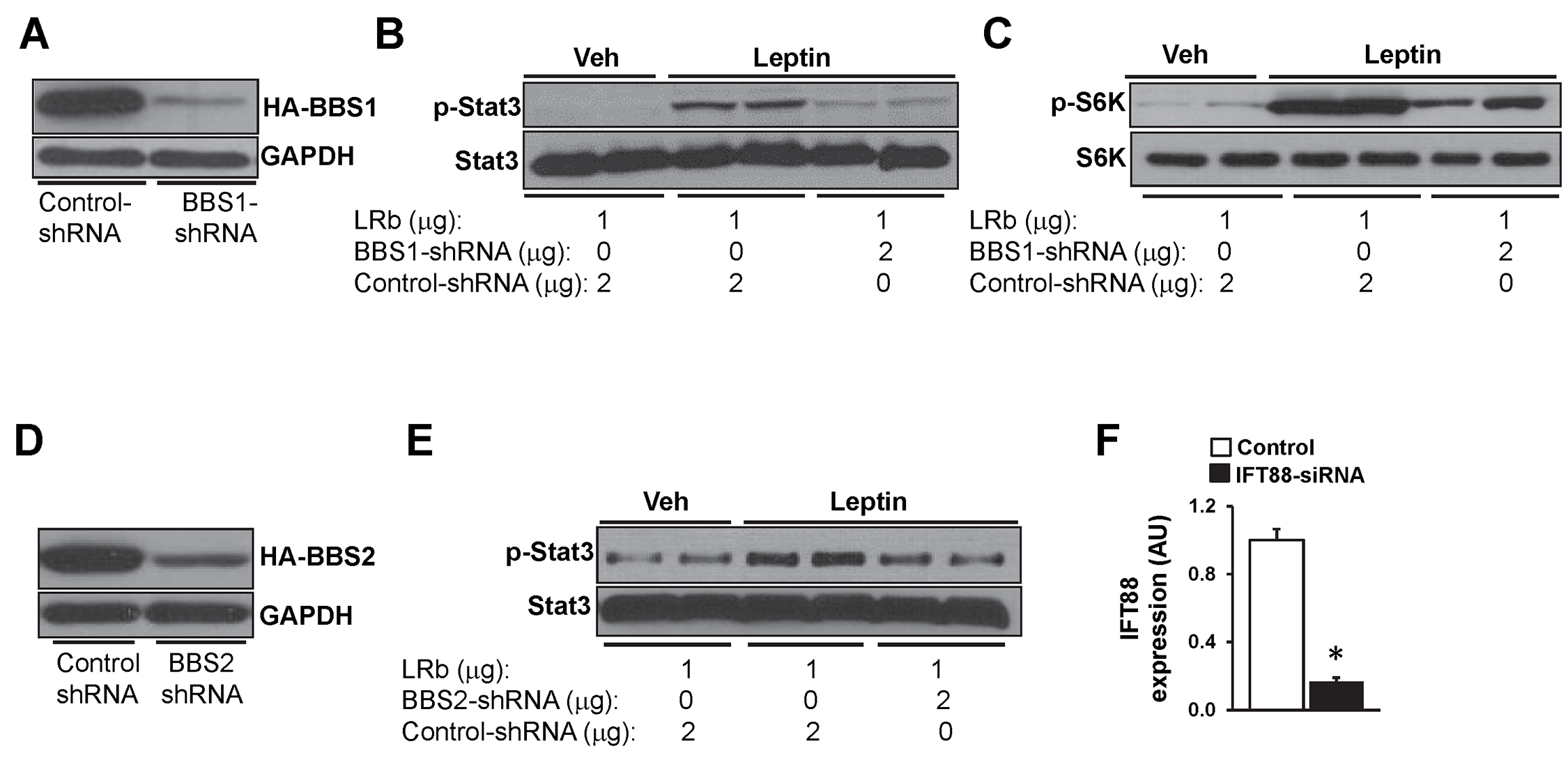

Supplement: S10 Fig — (A) Efficacy of BBS1-shRNA in HEK 293 cells to reduce HA-tagged BBS1 protein expression. (B—C) Knockdown of BBS1 attenuate leptin-induced activation of Stat3 (B) and S6K (C) in HEK 293 cells. (D) Efficacy of BBS2-shRNA in HEK 293 cells. (E) Knockdown of BBS2 attenuated leptin-induced activation of Stat3 in HEK 293 cells. (F) Efficacy of Ift88-siRNA in HEK 293 cells. Ift88 mRNA expression was measured by real-time RT-PCR (n = 4 per group). Data are means ± SEM. *P< 0.05 vs control group. (TIF) [file pgen.1005890.s010.tif]
