# Supplementary material for: The BBSome Controls Energy Homeostasis by Mediating the Transport of the Leptin Receptor to the Plasma Membrane
Source: PLoS Genet. 2016 Feb 29;12(2):e1005890. doi: 10.1371/journal.pgen.1005890 (PMC4771807; doi:10.1371/journal.pgen.1005890)
Supplement: S1 Table — (PDF) [file pgen.1005890.s011.pdf]

Name	Sense (5'-3')	Anti-sense (5'-3')
Genotyping		
Cre	ACCTGAAGATGTTCGCGATTATCT	ACCGTCAGTACGTGAGATATCTT
Bbs1	ACATCCACACCTTCTCCTCCT	GCCTACCTTGTACTCCCCATC
Ift88	GCCTCCTGTTTCTTGACAACAGTG	GGTCCTAACAAGTAAGCCCAGTGTTTC
td-Tomato	GGCATTAAAGCAGCGTATCC	CTGTTCCTGTACGGCATGC
Bbs1 gene expression in brain and peripheral tissues		
β–actin	CATCCTCTTCCTCCCTGGAGA	TTCCATACCCAAGAAGGAAGG
Bbs1	ACTGGCAGATTTGCATGGCGA	TCAGTGCCTAGCACCAGACAG
Bbs1 (qRT-PCR)	ACATCCACACCTTCTCCTCCT	CCAGCTTGTACTCCCCATCG
Genes encoding the BBSome and CLP proteins		
Bbs1	TTGTCTGTGCAGTCACTCAG	CGTCCTCATCTGCCAGGTT
Bbs2	GAGCTTCTTGTTGGATCTGAAGA	GACTGCCATACATAGGACACAG
Bbs4	CAAGCATGACCTGACTTACATAAT	CACACCAAGCTGCAAGTAGAGCA
Bbs5	GGAGCATGTGTATGATAAGAT	TCCTCCACTCTGCTGAGAGCTT
Bbs6	TGACTCTGATGGAATCTCTAAGC	TCTTGCAGAGCACACATCTTTCAC
Bbs7	TCAAAGCTGTCAACACACAAG	GTCTTAGCACTGCATAATGGA
Bbs8	CTATCACTAGCTCATCTGGAAG	ACATCGTTTTCATGGTGGAGAA
Bbs9	CCAGTGCTGCAAGTGGAAGTC	GGTTTCCATGTTCCACATTTC
Bbs10	CTACCAGAGGTTGCTTCACA	AGAGTGAGGTATAAATGCACATG
Bbs12	GGACAGATAATAGCCACTGG	CTTGCTGCAGACACAGACTCTG
Hypothalamic gene expression		
S18	ACTGCCATTAAGGGCGTGG	CCATCCTTCACATCCTTCTG
AgRP	CAGAAGCTTTGGCGGAGGT	AGGACTCGTGCAGCCTTACAC
NPY	TCAGACCTCTTAATGAAGGAAAGCA	GAGAACAAGTTTCATTTCCCATCA
POMC	CTGCTTCAGACCTCCATAGATGTG	CAGCGAGAGGTCGAGTTTGC
PTP1B	GACTCGTCAGTGCAGGATCA	GACTCGTCAGTGCAGGATCA
SOSC3	ACCAGCGCCACTTCTTCACG	GTGGAGCATCATACTGATCC
LR-RGRP	GTTTACTGGCCCCTGTTCGT	GGCTACCACTGTTCCCAACT
Silencing Ift88		
Ift88	GCCTCCTGTTTCTTGACAACAGTG	GGTCCTAACAAGTAAGCCCAGTGTTTC
hS18	ACTGCCATTAAGGGTGTGG	CCATCCTTTACATCCTTCTG
